# Insights Into the Peroxisomal Protein Inventory of Zebrafish

**DOI:** 10.3389/fphys.2022.822509

**Published:** 2022-02-28

**Authors:** Maki Kamoshita, Rechal Kumar, Marco Anteghini, Markus Kunze, Markus Islinger, Vítor Martins dos Santos, Michael Schrader

**Affiliations:** ^1^College of Life and Environmental Sciences, Biosciences, University of Exeter, Exeter, United Kingdom; ^2^LifeGlimmer GmbH, Berlin, Germany; ^3^Systems and Synthetic Biology, Wageningen University & Research, Wageningen, Netherlands; ^4^Center for Brain Research, Medical University of Vienna, Vienna, Austria; ^5^Institute of Neuroanatomy, Mannheim Center for Translational Neuroscience, Medical Faculty Mannheim, Heidelberg University, Mannheim, Germany

**Keywords:** peroxisomes, *Danio rerio*, proteome, lipid metabolism, organelle biogenesis, protein targeting, PTS1

## Abstract

Peroxisomes are ubiquitous, oxidative subcellular organelles with important functions in cellular lipid metabolism and redox homeostasis. Loss of peroxisomal functions causes severe disorders with developmental and neurological abnormalities. Zebrafish are emerging as an attractive vertebrate model to study peroxisomal disorders as well as cellular lipid metabolism. Here, we combined bioinformatics analyses with molecular cell biology and reveal the first comprehensive inventory of *Danio rerio* peroxisomal proteins, which we systematically compared with those of human peroxisomes. Through bioinformatics analysis of all PTS1-carrying proteins, we demonstrate that *D. rerio* lacks two well-known mammalian peroxisomal proteins (BAAT and ZADH2/PTGR3), but possesses a putative peroxisomal malate synthase (Mlsl) and verified differences in the presence of purine degrading enzymes. Furthermore, we revealed novel candidate peroxisomal proteins in *D. rerio*, whose function and localisation is discussed. Our findings confirm the suitability of zebrafish as a vertebrate model for peroxisome research and open possibilities for the study of novel peroxisomal candidate proteins in zebrafish and humans.

## Introduction

Peroxisomes represent ubiquitous, single-membrane bound subcellular compartments in eukaryotes. They are oxidative organelles with important functions in cellular lipid metabolism and redox homeostasis (Islinger et al., 2018). In mammals, peroxisomes perform a variety of essential metabolic functions including fatty acid α- and β-oxidation, degradation of D-amino acids, contribution to purine catabolism, and biosynthesis of ether lipids, polyunsaturated fatty acids and bile acids (Wanders and Waterham, 2006). Loss of peroxisomal functions causes severe disorders with developmental and neurological abnormalities, highlighting the importance of peroxisomes for human health. Loss of functional peroxisomes or enzyme deficiencies in humans result in an accumulation of undegraded molecules [e.g., very long chain fatty acids (VLCFA), bile acid intermediates, phytanic acid], while physiologically essential molecules (e.g., bile acids, plasmalogens, docosahexaenoic acid) become deficient. In addition to lipid metabolism, mammalian peroxisomes play a role in several non-lipid metabolic pathways such as purine, polyamine, glyoxylate, and amino acid metabolism. Due to their role in H_2_O_2_ metabolism and ROS homeostasis, peroxisomes have also been linked to cellular ageing and age-related disorders as well as cancer (Fransen et al., 2013; Islinger et al., 2018). Moreover, functions in cellular signalling and anti-viral defence have been revealed (Dixit et al., 2010; Lismont et al., 2019). However, the role of peroxisomes in the functioning of tissues and organs or during developmental processes remains largely unresolved.

The zebrafish (*Danio rerio*) is a popular vertebrate model organism for developmental biology and neurobiology due to its similarly to mammals. Advantages are the rapid development and short generation time, relatively low cost, ease of genetic manipulation and optical transparency of the developing fish, which in combination with novel imaging techniques allows the *in vivo* visualization of biological processes at the organism level (Dawes et al., 2020). In addition, zebrafish are used as a model to study lipid metabolism in lipid-related diseases (Hölttä-Vuori et al., 2010; Turchini et al., 2022). As peroxisomes are crucial for cellular lipid metabolism and developmental processes as well as neurological functions, zebrafish represent an attractive model to study peroxisome biology. In line with this, zebrafish has successfully been used as a model for adrenoleukodystrophy (ALD) (Strachan et al., 2017), a devastating disorder based on a defect in ABCD1, the peroxisomal transporter for VLCFA (Engelen et al., 2014). Very recently, a zebrafish model for Zellweger syndrome disorder (ZSD), a group of severe peroxisome biogenesis disorders caused by loss of peroxisome functions, has also been developed (Takashima et al., 2021).

Using diaminobenzidine (DAB)-cytochemistry for catalase, a classical peroxisomal marker, proxisomes were already visualised in the embryo and adult zebrafish by light- and electron microscopy (Braunbeck et al., 1990; Krysko et al., 2010). Similar to mammals and man, peroxisomes were most prominent in the liver, renal proximal tubules and the intestinal epithelium. Similarly to rodents, zebrafish hepatic peroxisomes respond to peroxisome proliferators with an increase in peroxisome number in liver when fish were exposed to fibrates or phthalate esters (Ortiz-Zarragoitia et al., 2006; Venkatachalam et al., 2012). In line with this, Peroxisome Proliferator-Activated Receptors (PPARs), nuclear hormone receptors, which regulate the expression of genes involved in lipid metabolism, have been identified in zebrafish (Den Broeder et al., 2015).

Peroxisomal matrix protein import is mediated by the peroxisomal import receptors PEX5 and PEX7, which bind to type-1 or type-2 peroxisomal targeting signals (PTS1 or PTS2) on cargo proteins in the cytosol. The PTS1 receptor PEX5 recognises a C-terminal tripeptide (SKL-type), whereas PEX7 recognises a nonapeptide within the N-terminus (Walter and Erdmann, 2019; Kunze, 2020). Several predictors have been developed to identify peroxisomal proteins, their PTS, and their sub-peroxisomal location (Kunze, 2018; Anteghini et al., 2021).

Despite growing interest, a comprehensive analyis of the *D. rerio* peroxisomal protein inventory and metabolic pathways associated with peroxisomes as well as of peroxisomal targeting signals is still missing. In this study, we combined bioinformatics analyses with molecular cell biology, and provide the first comprehensive inventory of peroxisomal proteins, their targeting signals and association with metabolic pathways in zebrafish. A comparison with *H. sapiens* gained new insights into the basic peroxisomal protein inventory shared among vertebrates and revealed novel candidate peroxisomal proteins and functions in *D. rerio*. We show that *D. rerio* peroxisomal functions do not vary considerably from those in humans confirming the suitability of zebrafish as a vertebrate model for peroxisome research. Our findings open possibilities for the study of novel peroxisomal candidate proteins in zebrafish and humans.

## Materials and Methods

### Search for Potential Peroxisomal Proteins in *Danio rerio*

**FIGURE 1:**
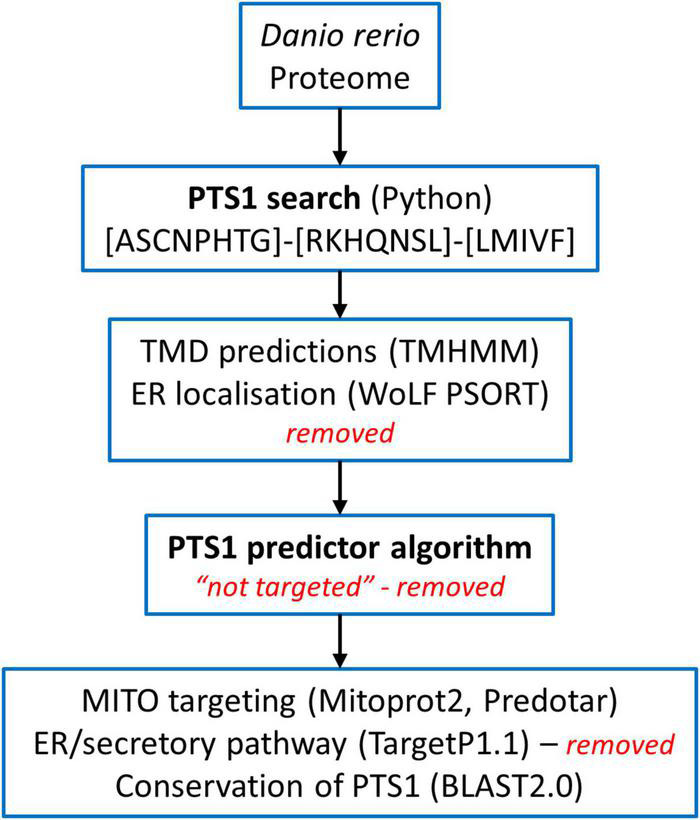
Overview of the screening of the *Danio rerio* proteome to identify candidate proteins with a C-terminal peroxisomal targeting sequence (PTS1). See Materials and Methods for details.

The *Danio rerio* proteome available on UniProt^1^ was screened for proteins carrying a PTS1 at the very C-terminus using all possible combinations of residues found in PTS1 motifs (consensus) [ASCNPHTG]-[RKHQNSL]-[LMIVF] (Lametschwandtner et al., 1998; Neuberger et al., 2003; Figure 1). Among 46,848 proteins, we identified 2,638 proteins matching the pattern. We analysed the corresponding fasta sequences using the software TMHMM Server v. 2.0^2^ (Krogh et al., 2001). The entries with defined topologies ‘o’ (out) or ‘i’ (in), without transmembrane helices, were kept as well as the entries with a ratio ≥ 0.9 between the expected number of amino acids in transmembrane helices and the expected number of amino acids in transmembrane helices in the first 60 amino acids of the protein (with transmembrane helices just in the signal peptide) resulting in 1,966 protein sequences (Figure 1). We executed locally WoLF PSORT (Package Command Line Version 0.2) (Horton et al., 2007) to obtain the predicted subcellular location of each specific protein. Entries with ‘‘ER’’ as possible subcellular localization were removed, resulting in 1,171 sequences. Thereafter, the identified proteins were further analysed by PTS1 predictor algorithms^3^ (Neuberger et al., 2003; Schlüter et al., 2010) and sequences which produced no hit with the “metazoa” or “general” modus of the software were removed (Figure 1). For further validation selected sequences were screened for conservation of the potential PTS1 using BLAST2.0. Additionally, mitochondrial targeting was examined using Mitoprot2 (Claros and Vincens, 1996); and Predotar1.03 (Small et al., 2004); potential targeting to the secretory pathway was screened with TargetP1.1 (Emanuelsson et al., 2000). Peroxisomal targeting signal 2 (PTS2) was analysed by PTS2 prediction algorithms (Schlüter et al., 2007). Functions were attributed to the potential peroxisomal proteins with regard to their homology to known proteins from other species and proteins were organised into specific metabolic pathways. The ZFIN zebrafish information network database^4^ was screened for peroxisomal proteins (e.g., without a PTS1) including PMPs and peroxisome biogenesis factors (peroxins), by key word search and BLAST analysis. Furthermore, published data (Pub Med) was included (e.g., Jansen et al., 2021). Protein sequence alignment was performed by Clustal Omega (1.2.4) Multiple Sequence Alignment (Madeira et al., 2019).

### Mammalian Cell Culture and Transfection

COS-7 (African green monkey kidney cells, CRL-1651; ATCC) and HEK293T (Human embryonic kidney 293T cells; ECACC) cells were maintained in Dulbecco’s Modified Eagle’s Medium (DMEM), high glucose (4.5 g/L) supplemented with 10% fetal bovine serum (FBS), 100 U/ml penicillin and 100 μg/ml streptomycin (all from Life Technologies) at 37°C with 5% CO2 and 95% humidity. COS-7 cells were transfected with DNA constructs either by incubation with diethylaminoethyl (DEAE)-dextran (Sigma-Aldrich) (Bonekamp et al., 2010) or Turbofect (Thermo Fisher Scientific) (Supplementary Table S1). For DEAE-transfection, 4 μg of plasmid DNA were mixed with 0.5 ml serum-free DMEM and 4 μg of DEAE–Dextran. The mixture was applied to a 60-mm dish of non-confluent cells after washing twice with PBS. After incubating 1.5 h at 37°C in a humidified incubator the DEAE–Dextran–DNA mixture was removed and 4 ml complete DMEM as well as 4 μl of chloroquine (Serva, Heidelberg, Germany) (60 mg/ml stock solution) were added. After 3 h the medium was changed to complete DMEM. For lipofection, 1 μg of DNA [0.1 μg EGFP-reporter plasmid and 0.9 μg vector DNA without EGFP (pcDNA3.1)] was premixed with 100 μl DMEM without supplements and 1.25 μl of Turbofect was added. After 15 min incubation at room temperature the mixture was dropped onto COS7 cells in 400 μl fresh complete DMEM medium (24-well plate). After 24 h cells were trypsinized and 50% of the cells were seeded on glass coverslips (24-well plate). Cells were processed 24-48 h after transfection for immunofluorescence microscopy.

### Immunofluorescence and Microscopy

Cells grown on glass coverslips were fixed for 20 min with 4% para-formaldehyde in PBS, pH 7.4, permeabilised with 0.2% Triton X-100 (10 min), blocked with 1% BSA (10 min) and sequentially incubated with primary and secondary antibodies for 1 h in a humid chamber at room temperature (Bonekamp et al., 2013). Rabbit anti-PEX14 (1:1400) (Grant et al., 2013) (generated by D. Crane, Griffith University, Brisbane, Australia), anti-PMP70 (1:3000) (ABR, Golden, CO, United States), and mouse anti-Myc primary antibody (1:100) [Santa Cruz Biotechnology, Inc (9E10)] were used. Species-specific Alexa Fluor 488 (594) labelled secondary antibodies (1:500) (Thermo Fisher Scientific) and Cy3-labelled Donkey-anti-rabbit IgG (1:400) (Jackson Immuno Research Laboratories, West Grove, PA, United States) were applied. Microscopy analysis was performed using an Olympus IX81 microscope (Olympus Optical. Hamburg, Germany) equipped with an UPlanSApo 100 × /1.40 oil objective (Olympus) and a CoolSNAP HQ2 CCD camera. Digital images were taken and processed using VisiView software (Visitron Systems). Images were adjusted for contrast and brightness using MetaMorph 7 (Molecular Devices).

### Molecular Cell Biology and Generation of Plasmids

For cloning of human genes, total RNA was extracted from HEK cells using Nucleo Spin RNA II kit (Macherey-Nagel- NZ74095550) and reverse transcribed into cDNA using SuperScript^®^ II Reverse Transcriptase kit (Invitrogen/Fisher). N-terminally Myc-tagged expression constructs for human CDC5L (NM_001253.4) (Cell division cycle 5-like protein) (Myc-*Hs*CDC5L) and KCTD5 (NM_018992.4) (Potassium channel tetramerization domain-containing 5) (Myc-*Hs*KCTD5) were generated. Human CDC5L (Q99459) and KCTD5 (Q9NXV2) were amplified from HEK cDNA, and the PCR products inserted into pCMV-Tag-3 vector (Agilent Technologies, La Jolla, CA, United States) (Supplementary Tables S1, S2).

To test the functionality of putative PTS1 or PTS2 motifs, reporter proteins were used. For PTS1 motifs the plasmid EGFP-C3 (Clontech) was digested with *Bgl*II/*Hin*dIII and the respective oligonucleotides were inserted as described previously (Chong et al., 2019; Supplementary Tables S1, S2). This generated plasmids PTS1-*Dr*Urad DLHSIVLSDIQTKL, PTS1-*Dr*Meox2a DLHDSDQSSDHAHL, PTS1-*Dr*Cdc5l DLLMLDKQTLSSKI, PTS1-*Dr*Kctd5a DLKAKILQEQGSRM, and PTS1-*Hs*CDC5L DLLLEKETLKSKF. For PTS2 motifs the reporter plasmid PTS2-tester (Kunze et al., 2011) was digested with *Eco*RI and *Pst*I and the oligonucleotides (Oli_2982 and Oli_2983) were inserted to generate PTS2-*Dr*Urah RLQHIRGHI (Supplementary Tables S1, S2). In-frame insertion of all constructs was verified by sequencing (Eurofins Genomics).

## Results and Discussion

**FIGURE 2:**
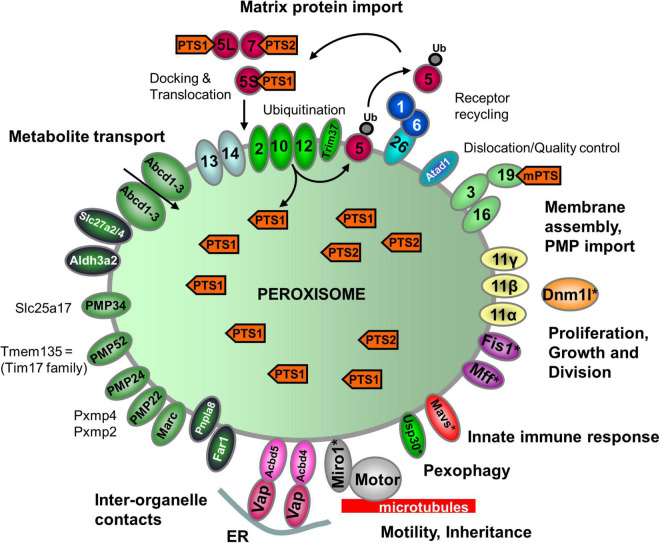
Schematic overview of the predicted molecular machineries and *D. rerio* proteins localized at the membranes of peroxisomes in zebrafish. See text for further details. *Matrix protein import*: after synthesis on free ribosomes, cargo proteins containing the peroxisomal targeting signals PTS1 or PTS2 bind to the corresponding cytosolic receptors Pex5 (E7FGF7) or Pex7 (A8KBW8) (Pex-proteins are indicated as sole numbers) and form receptor–cargo complexes. The Pex7–cargo complex requires accessory factors for import (e.g., Pex5L, a long isoform of Pex5). Import is achieved by a complex set of integral or peripheral PMPs that form the matrix protein import machinery, which mediates docking of the cargo-bound import receptor at the peroxisomal membrane [Pex13 (Q6PFQ3), Pex14 (A0A2R8QMZ2)], cargo translocation into the matrix of the organelle by a dynamic translocon [Pex2 (E7F4V8), Pex10 (Q5XJ92), Pex12 (B0R157)], and export of the receptor back to the cytosol [Pex1 (A0A0R4IPF0), Pex6 (F1QMB0)]. Recycling of the receptor involves its ubiquitination (Ub) and extraction from the membrane by an AAA–ATPase complex (Pex1, Pex6). Pex6 binds to the membrane protein Pex26 (F1RBL0). *Membrane assembly and insertion of PMPs* (containing an mPTS) depend on Pex19 (F1R313), Pex3 (Q5RIV3), and Pex16 (F1RDG2). Pex19 functions as a cycling receptor/chaperone, which binds the PMPs in the cytosol and interacts with Pex3 at the peroxisomal membrane. *Proliferation, growth and division*: Pex11α (A3QJY9), Pex11β (Q0P453), and Pex11γ (Q4V8Z0) are involved in the regulation of peroxisome size and number (proliferation). Pex11β remodels the peroxisomal membrane and interacts with the membrane adaptors Mff (F1Q877; A8E7S0) and Fis1 (A0A2R8Q8G0), which recruit the dynamin 1-like fission GTPase Dnm1l to peroxisomes, which in mammals is activated by Pex11β. *Motility*: mammalian peroxisomes move along microtubules, and Miro/Rhot (Rhot1a, Q6NVC5; Rhot1b, A0A0R4IGX0; Rhot2, Q32LU1) serves as membrane adaptor for the microtubule-dependent motor proteins kinesin and dynein. *Tethering*: Acbd5 (E9QCH6; A5WV69) and Acbd4 (F1QA31) interact with ER-resident Vap (Vapb, Q6P2B0) to mediate peroxisome–ER contacts. *Metabolite transport*: uptake of fatty acids in *D. rerio* peroxisomes is mediated by ABC transporter proteins (Abcd1, F1RBC8; Abcd2, E7F973; Abcd3a, A0A0R4IRL4; Abcd3b, B0UY91). Other peroxisomal transporter and membrane proteins in zebrafish include (functions are in part unclear): PMP34 (Slc25a17) (A5D6T2), a peroxisomal CoA transporter; PMP52 (Tmem135) (A4QN71) and PMP24 (Pxmp4) (A0A2R8QFW3) belong to the Tim17 family of transporters; PMP22 (Pxmp2) (Q66HU7); Slc27a2/4 (F1QQC5, Q1ECW0; Q567D7), acyl-CoA synthetase long chain family member; Marc (A0A2R8PWS6), mitochondrial amidoxime reducing component; Atad1 (A0A2R8RM20, B2GP29), ATPase family AAA (ATPase associated with various cellular activities) domain-containing protein 1 with a potential role in dislocation/quality control of tail-anchored membrane proteins; Aldh3a2 (A0A2R8PW97, E9QH31), fatty aldehyde dehydrogenase; Far1/2 (A0A0R4ICF6/Q1L8Q4), fatty acyl-CoA reductase 1/2 (ether lipid biosynthesis); Mavs (F1REK4), mitochondrial antiviral signalling protein with a putative role in innate immune response; Trim37 (E7FBZ8), tripartite motif-containing protein 37, an E3 ubiquitin-protein ligase involved in Pex5 mediated peroxisomal matrix protein import; Usp30 (A2BGT0), ubiquitin-specific protease 30, a deubiquitinase involved in the turnover of peroxisomes; Pnpla8 (F1RE62), Patatin-like phospholipase domain-containing 8. Proteins with a potential dual localization to both peroxisomes and mitochondria are marked with an asterisk. Pex, peroxin; PMP, peroxisomal membrane protein (adapted from Islinger et al., 2018, but containing the zebrafish specific nomenclature).

The study of model organisms has greatly contributed to our understanding of peroxisome biology. Fungal model systems greatly contributed to the identification of peroxisome biogenesis factors (peroxins, PEX), and to the understanding of protein import and peroxisome functions. The fruitfly *Drosophila melanogaster* has also been established as a non-vertebrate model organism to study peroxisomes (Pridie et al., 2020). In addition, several mouse models have been developed to investigate the physiological role of peroxisomes and their impact on human disease (Van Veldhoven and Baes, 2013). The zebrafish *Danio rerio* is now developing as a promising vertebrate model to investigate peroxisome biology (Van Veldhoven and Baes, 2013), but a comprehensive analysis of the *D. rerio* peroxisomal protein inventory, metabolic pathways and protein targeting is lacking. To delineate the peroxisomal proteome of *D. rerio* as well as targeting information of candidate proteins, we performed a comprehensive bioinformatics analysis (Figure 1). For identification of proteins with a potential peroxisomal targeting signal, we screened the proteome of *D. rerio*^5^ for proteins with a PTS1 or PTS2 sequence (PTS2 based on orthologous protein sequences). These are recognised by the matrix protein import receptors PEX5 and PEX7, respectively (Figure 2). The initial PTS1 search was performed with the broader consensus [ASCNPHTG]-[RKHQNSL]-[LMIVF] (Lametschwandtner et al., 1998; Neuberger et al., 2003) to also detect potential non-canonical C-terminal PTS1 sequences (and resulted in 2,638 proteins). We then determined potential ER targeting signals as well as the presence of transmembrane domains (TMD). Proteins with an ER signal peptide or TMD were excluded resulting in 1171 proteins. The identified candidate proteins were afterward analysed by PTS1 predictor algorithms, which consider 12 aa at the C-terminus. This approach resulted in a total of 371 candidate proteins with a potential PTS1 (Supplementary Tables S3, S4). Of those, 204 had a predicted weak targeting signal (twilight zone). We attributed functions to the candidate proteins based on homology to known proteins from other species and organised them into specific metabolic pathways. This was in part supported by literature and database search (ZFIN; PODB) (Table 1 and Supplementary Table S3). Peroxisomal membrane proteins and peroxins were identified by key word and database search as well as BLAST analysis.

**TABLE 1:** Inventory of *Danio rerio* peroxisomal proteins and metabolic pathways in comparison to *H. sapiens*.

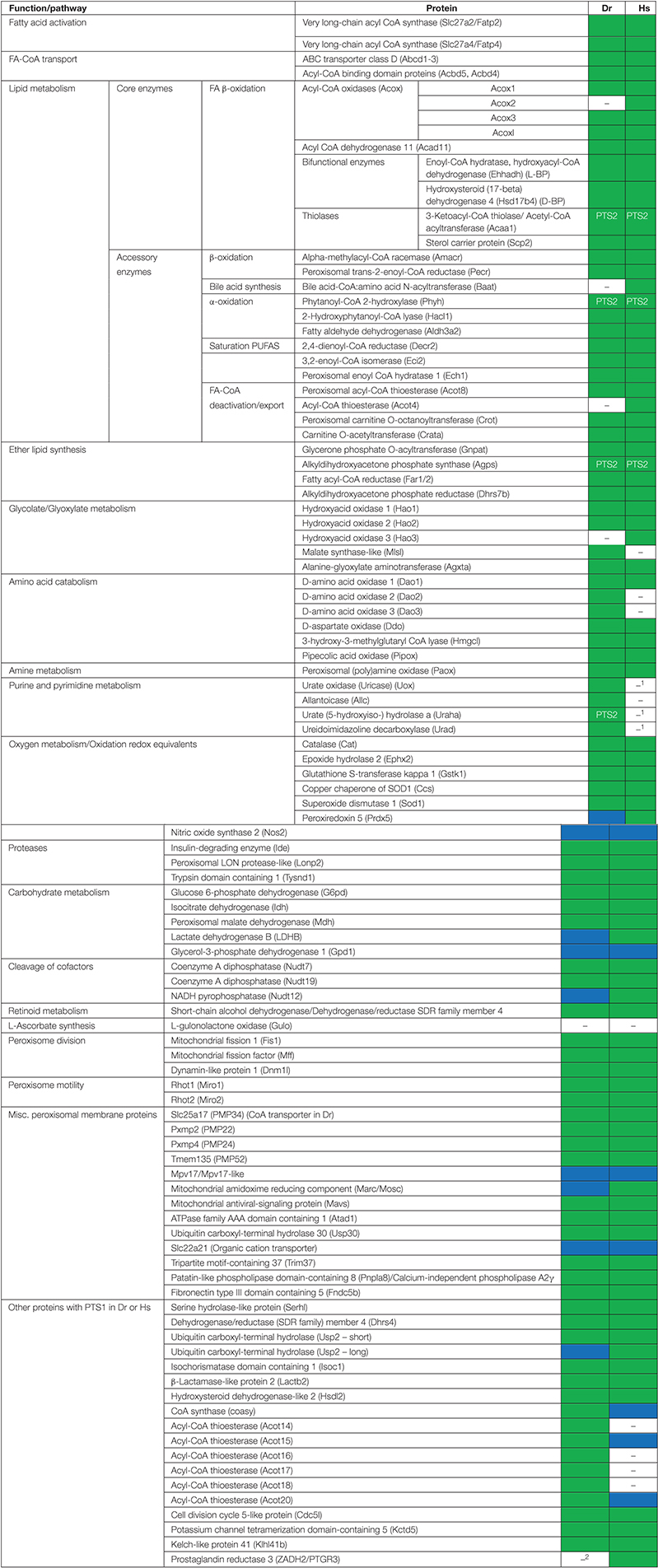

*Hs, H. sapiens; Dr, D. rerio.*

*□ – absent/not identified.*

*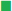 – Protein present, peroxisomal and/or with a predicted PTS1/PTS2.*

*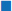 – Protein present without a confirmed PTS or peroxisomal localization.*

*^1^inactivation by pseudogenization.*

*^2^lost in Cypriniformes.*

### Comparison of the Classical Peroxisomal Protein Inventory Shared by Humans and Zebrafish

In the following, we present the results of our comprehensive analysis of the *D. rerio* peroxisomal protein inventory and metabolic pathways associated with peroxisomes. First, we present and discuss the “core” peroxisomal proteins involved in biogenesis, dynamics and metabolic pathways including fatty acid oxidation, ether lipid biosynthesis, purine catabolism and ROS metabolism. Furthermore, we link our analyses to recent publications on peroxisome research in zebrafish providing a timely overview.

### Peroxin Proteins

Peroxins (encoded by PEX genes) represent proteins essential for the biogenesis of peroxisomes. They include PEX proteins required for peroxisomal matrix protein import, membrane biogenesis, and peroxisome proliferation. *D. rerio* encodes orthologues of all 14 human peroxins (Figure 2 and Supplementary Table S3). They belong to the core of PEX proteins that are broadly conserved in most eukaryotes, including PEX3/16/19 (peroxisomal membrane protein sorting), PEX1/6, PEX2/10/12, PEX13/14, and PEX5/7 (matrix protein import) and proteins of the PEX11 family (peroxisome proliferation) (Figure 2 and Supplementary Table S3). Overall, 37 peroxins have been identified in yeast, plants and animals (including functional orthologues). Being a vertebrate, *D. rerio* most closely reflects the situation in humans. Interestingly, *Dr*Pex3 possesses a predicted N-terminal mitochondrial targeting signal (MTS) (Figure 2 and Supplementary Table S3). Potentially hidden N-terminal MTS are also found in human PEX3 and may explain its mistargeting to mitochondria in the absence of peroxisomes (Sugiura et al., 2017). Like *H. sapiens*, *D. rerio* encodes three isoforms of Pex11 (Pex11a, Pex11b, Pex11g), which in mammals are involved in the growth, division and proliferation of peroxisomes. In addition to the PTS1 receptor Pex5, two Pex5-related proteins (Pex5la; Pex5lb) are present in *D. rerio*. It should be noted that zebrafish often harbour two copies of many genes. This is due to a genome wide duplication event, which took place approx. 350 million years ago when the bony fishes diverged from the common ancestor with humans (Hölttä-Vuori et al., 2010). The duplicated genes often exhibit differential tissue expression patterns with partitioning of ancestral functions, rather than the evolution of completely new functions (Force et al., 1999). The Pex5-related proteins may represent paralogs of Pex5, which may no longer function in peroxisomal matrix protein import. PEX5-related proteins are found in other vertebrates; PEX5R/TRIP8b (tetratrico-peptide-repeat containing, Rab8b-interacting protein) is involved in the regulation of hyperpolarization-activated cyclic nucleotide-gated (HCN) channels in the mammalian central nervous system (Han et al., 2020). Although PEX5R can bind PTS1-containing proteins *in vitro* (Amery et al., 2001), there is currently no evidence for a role in peroxisome biogenesis as PEX5R does not complement loss of PEX5. The PTS1 receptor Pex5 of *D. rerio* contains several characteristic tetratricopeptide repeats (TPR) at the C-terminus involved in the interaction with the PTS1 cargo, and a disordered region at the N-terminus. The latter contains a PEX7-binding domain which is conserved in PEX5 proteins of several species (e.g., PEX5L, the long isoform in humans) (Figure 2 and Supplementary Figure S1; Jansen et al., 2021) and enables function as a PEX7 co-receptor for PTS2 import (Kunze, 2020). This indicates that Pex5 is required as a co-receptor for Pex7-mediated PTS2 import in *D. rerio*.

While Pex7 (and PTS2 cargo proteins) are present in *D. rerio*, other model organisms such as the nematode *C. elegans*, lack PEX7 and a PTS2 targeting pathway (Motley et al., 2000). A PTS2 import pathway is also lacking in the fruit fly *D. melanogaster*, but PEX7 is present (Pridie et al., 2020). *C. elegans* and *D. melanogaster* also appear to lack PEX26, the anchoring protein for PEX1/6, which is present in *D. rerio* and other vertebrates (Jansen et al., 2021). The AAA (ATPase associated with diverse cellular activities) ATPases PEX1 (Pex1, A0A0R4IPF0_DANRE) and PEX6 (Pex6, F1QMB0_DANRE) are required for PEX5 export from the peroxisomal membrane in order to recycle it back to the cytosol. Like in humans, *D. rerio* Pex26 is a tail-anchored membrane protein supposed to retain Pex1 and Pex6 at the membrane (Figure 2 and Supplementary Table S3).

Very recently, a zebrafish model for Zellweger spectrum disorders, a group of severe peroxisome biogenesis disorders based on defects in PEX genes has been developed (Takashima et al., 2021). Disruption of the zebrafish *pex2* gene (encoding an E3 ubiquitin ligase with a zinc RING finger domain residing in the peroxisomal membrane, which is involved in matrix protein import/receptor recycling) caused phenotypes similar to human patients suffering from ZSD including locomotive defects, eating disabilities, liver abnormalities and early death (Takashima et al., 2021). Similar to the human disease, the ZS model fish also showed increased tissue levels of VLCFA and branched chain fatty acids as well as a reduction in ether phospholipids. Furthermore, mutant specific gene-expression changes, that might lead to the symptoms, were detected including a reduction in crystallin (lens), troponin, parvalbumin (muscle contraction), and fatty acid metabolic genes.

### Proteins Involved in Peroxisome Division and Motility

In addition to PEX11 proteins, which are involved in membrane remodelling and growth/expansion of the peroxisomal membrane prior to division, the tail-anchored adaptor proteins FIS1 and MFF are required, which recruit the fission GTPase DRP1/DLP1 to the peroxisomal membrane (Schrader et al., 2016; Figure 2). Homologues of those proteins have been identified in *D. rerio* (Fis1, Mffa, Mffb, Dnm1l) (Table 1 and Supplementary Table S3). Interestingly, they are shared with mitochondria and also mediate mitochondrial division. In addition, the orthologues of the tail-anchored adaptor proteins Miro1/2 (Rhot1a, Rhot1b, Rhot2) are present in *D. rerio*. In mammals they have been implicated in the recruitment of microtubule motor proteins (e.g., kinesin) to peroxisomes (and mitochondria) and regulation of organelle motility. The Miro-motor complex can exert pulling forces at the peroxisome membrane, which also contribute to membrane elongation/expansion and division (Castro et al., 2018; Covill-Cooke et al., 2021). Targeting of the tail-anchored membrane proteins to peroxisomes is mediated by a combination of biochemical properties of the TMD and tail region (TMD hydrophobicity; positive net charge of the tail) and depends on PEX19, the import receptor/chaperone for PMPs (Costello et al., 2017a; Figure 2). In addition to its role in matrix protein import, PEX14 may act as docking factor to microtubules via its N-terminal tubulin binding domain (aa 1-78) (Reuter et al., 2021), which is conserved between *H. sapiens* and *D. rerio*.

### Peroxisomal Metabolism

#### Fatty Acid β-Oxidation

**FIGURE 3:**
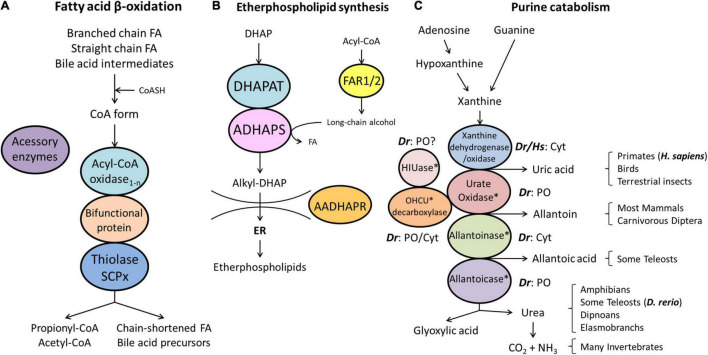
Schematic representation of the pathways of fatty acid β-oxidation **(A)**, ether phospholipid synthesis **(B)** and purine catabolism **(C)** in peroxisomes. **(A)** Peroxisomes degrade fatty acids in four consecutive steps: (1) oxidation, (2) hydration, (3) dehydrogenation, and (4) thiolytic cleavage. Step 1 is performed by multiple acyl-CoA oxidases (1-n) with different substrate specificities. Steps 2 and 3 involve two bifunctional proteins (L-BP, D-BP) harbouring both enoyl-CoA hydratase and 3-hydroxy-acyl-CoA dehydrogenase activity. Step 4 requires different thiolases (e.g., ACAA1, SCPx). Peroxisomes have acquired a set of accessory enzymes (e.g., for fatty acid α-oxidation) to transform the acyl-CoA esters of those fatty acids (FA), which cannot directly enter the β-oxidation pathway (e.g., phytanic acid) (see text for details). **(B)** The first three steps of ether phospholipid synthesis take place in peroxisomes; synthesis continues in the endoplasmic reticulum (ER). **(C)** Enzymes involved in purine catabolism in different vertebrate groups and their excretion products. Note that the cellular localisation (cytosolic, peroxisomal) of these enzymes varies amongst vertebrate species. Xanthine oxidase is cytosolic (Cyt) in *H. sapiens* (*Hs*) and *D. rerio* (*Dr*). Urate oxidase, HIUase, OHCU decarboxylase, and allantoicase are supposed to localise to peroxisomes (PO) in *D. rerio*, but are absent in *H. sapiens* (asterisks) (see also Table 1). Allantoinase is cytosolic in *D. rerio*, but absent in *H. sapiens* (asterisks). AADHAPR, alkyl-dihydroxyacetone phosphate reductase; ADHAPS, alkyl-dihydroxyacetone phosphate synthase; DHAP, dihydroxyacetone phosphate; DHAPAT, dihydroxyacetone phosphate acyltransferase; FAR1/2, fatty acyl-CoA reductases 1/2; HIUase, 5-hydroxyisourate hydrolase; OHCU decarboxylase, 2-oxo-4-hydroxy-4-carboxy-5-ureidoimidazoline decarboxylase (adapted from Islinger et al., 2010).

Peroxisomes fulfil important functions in cellular lipid metabolism, often in cooperation with other subcellular compartments such as mitochondria, ER and lipid droplets (Silva et al., 2020). The β-oxidation of fatty acids is an important and conserved peroxisomal pathway (see overview Figure 3A). Fatty acid β-oxidation is distributed between peroxisomes and mitochondria in animals and several fungi, but exclusively peroxisomal in yeast and plants. In line with this, we identified candidate genes coding for all the enzymes required for a peroxisomal and a mitochondrial fatty acid β-oxidation pathway in *D. rerio* (Table 1 and Supplementary Table S3). Substrates for peroxisomal β-oxidation are VLCFA (>C22:0), branched chain fatty acids (e.g., pristanic acid), bile acid intermediates [such as di- and tri-hydroxycholestanoyl-CoA (DHCA, THCA)], poly-unsaturated fatty acid (e.g., docosahexaenoic acid), long chain dicarboxylic acids, 2-hydroxy fatty acids, and a number of prostanoids. Mitochondria on the other hand preferentially β-oxidise long chain, medium chain, and short chain fatty acids (C18 and shorter).

Fatty acid degradation is preceded by activation of fatty acids through conjugation to coenzyme A and subsequent import into peroxisomes by members of the ATP-binding cassette (ABC) transporter subfamily D (reviewed in Chornyi et al., 2020; Figure 2). The esterification of fatty acids into their corresponding CoA esters is catalysed by acyl-CoA synthetases, and is a requirement for import via the peroxisomal ABCD transporters as well as for fatty acid β-oxidation. In humans, the two very-long-chain acyl-CoA synthetases SLC27A2 (FATP2) and SLC27A4 (FATP4) have been partially localized to the peroxisomal membrane (reviewed in Chornyi et al., 2020). Both proteins are mainly involved in the activation of fatty acids rather than transport across membranes. Similar to human SLC27A2, the two *D. rerio* orthologues (Slc27a2a, Slc27a2b) have a non-canonical PTS1 (TRL, FRL, respectively) (Table 1 and Supplementary Table S3). The *D. rerio* Slc27a4 also possesses a non-canonical PTS1 (QKL) (Supplementary Table S3). PTS1 signals normally mediate the targeting of matrix proteins, and it has not been demonstrated that PTS1 signals also target membrane proteins like SLC27A2 and SLC27A4. However, peroxisomal SLC27A2 is supposed to be a peripheral membrane protein facing the matrix (Smith et al., 2000), and splice variants of SLC27A4, a putative transmembrane protein, with and without TMD may exist. It is discussed whether hydrolysis of the CoA ester bond is required for ABCD transporter-mediated acyl-CoA transport into peroxisomes (reviewed in Chornyi et al., 2020). In such a scenario, acyl-CoA synthetases must also be present in the peroxisomal matrix, e.g., to re-activate the imported fatty acids prior to β-oxidation. We also identified a *D. rerio* acyl-CoA synthetase long chain family member 5 (Acsl5, F1RAK0_DANRE) with a weak PTS1 (ANM), which appears to be an N-terminal tail-anchored membrane protein localising to multiple organelles (Supplementary Table S3). Mammalian ACSL1, the most abundant acyl-CoA synthetase in liver, is according to numerous proteomics studies also supposed to be peroxisomal, and possesses a TMD at the N-terminus (Watkins and Ellis, 2012; Yifrach et al., 2018).

In *D. rerio* four transporters homologous to the mammalian peroxisomal transporters ABCD1 (Abcd1, F1RBC8_DANRE), ABCD2 (Abcd2, E7F973_DANRE), and ABCD3 (Abcd3a, A0A0R4IRL4_DANRE; Abcd3b, B0UY91_DANRE) were identified (Figure 2, Table 1, and Supplementary Table S3). Defects in human ABCD1, required for the uptake of very long-chain fatty acids (VLCFA) into peroxisomes, cause adrenoleukodystrophy (ALD), a devastating neurodegenerative disease with central and peripheral demyelination (Engelen et al., 2014). It has been shown that *D. rerio* Abcd1 is highly conserved at the amino acid level with human ABCD1, and during development is expressed in corresponding regions/tissues including the central nervous system (CNS) and adrenal glands (Strachan et al., 2017). A zebrafish model of ALD has recently been established that recapitulates key features of the human disease pathology. Similar to ALD patients, zebrafish *abcd1* mutants have elevated VLCFA levels, develop a motor impairment, and show reduced life expectancy. Furthermore, CNS development was disrupted, with reduced numbers of oligodendrocytes with altered patterning, hypomyelination, and increased apoptosis (Strachan et al., 2017). The zebrafish ALD model has been successfully used in a drug screen to identify compounds to alleviate lipid toxicity (Raas et al., 2021).

In mammals, ABCD1 and ABCD2 are involved in the transport of long and very long chain fatty acids, whereas ABCD3 is suggested to mediate the transport of branched chain acyl-CoA, the bile acid intermediates di- and tri-hydroxycholestanoyl-CoA (DHCA and THCA), and medium to long-chain acyl-CoA (Violante et al., 2019). Under normal conditions, the latter are preferentially degraded in mitochondria. ABCD3 defects have been linked to hepatosplenomegaly, a liver disease (Ferdinandusse et al., 2015). ABCD4 (Abcd4, K9M7F0_DANRE), the fourth member of the ABC subfamily D, is no longer considered a peroxisomal protein. It localises to the ER and lysosomes, and is involved in vitamin B12 transport with defects leading to vitamin B12-deficiency anaemia in man and zebrafish (Kawaguchi and Morita, 2016; Choi et al., 2019).

In addition to the ABC transporters, the acyl-CoA binding domain containing proteins Acbd5a, Acbd5b, and Acbd4 have been identified in *D. rerio* (Figure 2, Table 1, and Supplementary Table S3). Human ACBD5 is required for VLCFA-acyl-CoA import via ABCD1, and its loss results in a new peroxisomal disorder, ACBD5 deficiency, with accumulation of VLCFA and neurological abnormalities (Ferdinandusse et al., 2017; Yagita et al., 2017; Darwisch et al., 2020). Furthermore, in mammals ACBD5 and ACBD4 are involved in the tethering of peroxisomes to the ER and membrane contact site formation (Costello et al., 2017b,c; Hua et al., 2017). Like human ACBD4/5, the zebrafish proteins are also tail-anchored membrane proteins.

Similar to mitochondrial β-oxidation, the peroxisomal pathway degrades fatty acids in four consecutive steps, namely by (i) oxidation, (ii) hydration, (iii) dehydrogenation, and (iv) thiolytic cleavage (Figure 3A). The first step involves acyl-CoA oxidases (ACOX), and we identified three ACOX in *D. rerio*: Acox1 (F1R071_DANRE), Acox3 (F1QXK3_DANRE) and an ACOX-like protein (Acoxl, A0A2R8QDD9_DANRE; F1R4J4_DANRE) (Table 1 and Supplementary Table S3). Alternative splicing isoforms of zebrafish Acox1 were described suggesting tissue-specific modulation of Acox1 activity (Morais et al., 2007). Acox1, Acox3 and Acoxl display canonical PTS1 (SKL, AKL, SKL), whereas the other Acoxl isoform (F1R4J4_DANRE) lacks a PTS1 or PTS2. Humans have four acyl-CoA oxidases (ACOX1, ACOX2, ACOX3, ACOXL/ACOX4), with ACOX1 preferentially degrading straight-chain fatty acids with different chain lengths, while ACOX2 is the only human acyl-CoA oxidase involved in bile acid biosynthesis, and both ACOX2 and ACOX3 are involved in the degradation of branched-chain fatty acids (Ferdinandusse et al., 2018). We noticed that for human ACOXL/ACOX4, which is not well studied (Van Veldhoven, 2010) similar to *D. rerio* Acoxl, a C-terminally extended isoform exists (Q9NUZ1-4), which possesses a PTS1 (AKL). If the other ACOXL/Acoxl isoforms lacking a PTS1 also localise to peroxisomes, e.g., in a complex with the PTS1-targeted isoforms, remains to be elucidated.

In animals, the second and third step is mediated by D- and L-bifunctional enzymes (D- and L-BP), which both function concomitantly as an enoyl-CoA hydratase and a 3-hydroxyacyl CoA dehydrogenase, but display different substrate specificity (Figure 3A). Both are present in *D. rerio* [Hsd17b4 (Dbp), Ehhadh (Lbp)] and contain a PTS1 (Table 1 and Supplementary Table S3). Knockdown of Dbp in zebrafish resulted in defective craniofacial morphogenesis, growth retardation, and abnormal neuronal development similar to D-BP mutations in humans (Kim et al., 2014). Furthermore, the development of blood, blood vessels, and endoderm-derived organs (e.g., liver, pancreas) was impaired suggesting that zebrafish is a useful model to study the role of peroxisomes during vertebrate development (Kim et al., 2014).

In humans, two peroxisomal thiolases, 3-ketoacyl-CoA-thiolase 1 (pTH1, ACAA1) and SCPx (sterol carrier protein x; pTH2), can exert the last step. pTH1 metabolizes only straight chain fatty acids, whereas the branched chain fatty acids and bile acid precursors (pristanic acid, DHCA, and THCA) are solely cleaved by SCPx. Sterol carrier protein 2 (SCP2) is a second isoform from the same gene lacking the N-terminal thiolase domain. SCP2 is a multilocalised protein described in peroxisomes, the cytosol, the ER and potentially mitochondria as it harbours a putative MTS at its N-terminus. *D. rerio* possesses a 3-ketoacyl-CoA thiolase (Acaa1) carrying a PTS2 as well as two sterol carrier proteins (Scp2a, Scp2b) with a PTS1 (AKL) (Table 1 and Supplementary Table S3). Scp2a represents the traditional SCPx including the thiolase domain (538aa), whereas Scp2b represents a shorter form (142aa) lacking the thiolase domain. The crystal structure of the zebrafish Scp2-thiolase was recently revealed (Kiema et al., 2019). Expression changes of *scp2a* and *acaa1*, as well as the β-oxidation genes *ehhadh* (L-BP), *hsd17b4* (D-BP), *acox1* and *acox3* were recently reported in a zebrafish model for *pex2* deficiency (Takashima et al., 2021).

The above mentioned proteins are involved in the β-oxidation of straight-chain saturated fatty acids and α-methyl branched-chain fatty acids with the methyl group in the (2S)-configuration (Wanders and Waterham, 2006). However, the β-oxidation of (2R)-methyl branched-chain FAs and unsaturated FAs requires auxiliary enzymes (Table 1). As direct oxidation of (2R)-methyl branched-chain fatty acids is not possible, the peroxisomal 2-methylacyl-CoA racemase (AMACR), which converts (2R)- into (2S) branched-chain acyl-CoAs, is required. The *D. rerio* 2-methylacyl-CoA racemase (Amacr) possesses a PTS1 (ARL) (Supplementary Table S3) and a potential N-terminal mitochondrial targeting signal (Amery et al., 2000).

Peroxisomes also contain enzymes to remove the double bonds in mono- and polyunsaturated fatty acids (Wanders and Waterham, 2006; Chornyi et al., 2020). Degradation of fatty acids with an internal double bond at an even-numbered position involves a state of two conjugated double bonds, which are subsequently processed by 2,4-dienoyl-CoA reductase (DECR2) and Δ3,Δ2-enoyl-CoA isomerase (PECI) before re-entering β-oxidation in the hydratase step. Both enzymes (Decr2, Eci2) are present in zebrafish both containing a PTS1 (Table 1 and Supplementary Table S3). Like the human enzyme, the *D. rerio* Δ3,5, Δ2,4-dienoyl-CoA isomerase (Ech1) is likely involved in the processing of fatty acids with a double bond at an odd-numbered position, and possesses a canonical PTS1 (SKL) (Table 1 and Supplementary Table S3). Furthermore, an additional gene encoding a potential enoyl-CoA isomerase/hydratase (zgc:101569, F1R2G5_DANRE) with a PTS1 (SKL) was identified (Supplementary Table S3).

After shortening of the fatty acid chains to medium chain fatty acyl-CoA by the peroxisomal β-oxidation pathway, the fatty acids are conjugated to carnitine, exit the peroxisomes, and undergo further β-oxidation in mitochondria (Houten et al., 2020). The *D. rerio* carnitine O-acetyltransferase a (Crata) displays a PTS1 (AKL), whereas the carnitine O-acetyltransferase b (Cratb) does not possess a PTS1. *D. rerio* carnitine octanoyltransferase (Crot) has a weak PTS1 (SQL) (Table 1 and Supplementary Table S3). Crot is likely involved in the export of the medium chain fatty acids, whereas Crat is most probably involved in the export of the acetyl-CoA units generated by β-oxidation.

**FIGURE 4:**
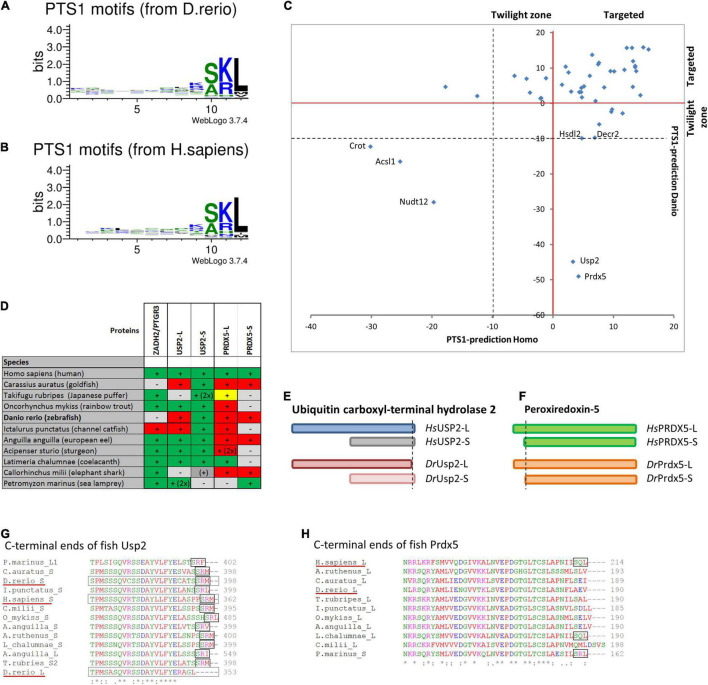
Comparison of PTS1 motifs between *D. rerio* and *H. sapiens*: To depict the difference in the average abundance of amino acids at different positions of the PTS1 in fish **(A)** and humans **(B)**, we selected proteins from each species, which were predicted to harbour a PTS1 (Neuberger et al., 2003), and depicted them as WebLogo (http://weblogo.threeplusone.com/create.cgi). **(C)** A comparison of the numerical values of the PTS1-predictions for those proteins is plotted for *H. sapiens* (*x*-axis) and *D. rerio* (*y*-axis) and depicted relative to the threshold for the evaluation of targeted proteins (>0, red) or the “twilight zone” (–10 < x < 0, grey); proteins with negative prediction are highlighted. **(D)** Systematic overview of orthologous fish proteins, which were either not found in *D. rerio* (ZADH2/PTGR3) or lack a PTS1 (Usp-L, long isoform; Prdx-5). (+) or (–) indicate presence or absence of the protein; 2x indicates 2 variants; traditional PTS1 identified (green) or not found (red); non-canonical PTS1 (yellow). **(E)** Schematic representation of the two isoforms of ubiquitin carboxyl-terminal hydrolase 2 (USP2) in *H. sapiens* (blue) and *D. rerio* (red). No PTS1 was found in the long isoform of Usp2 (Usp2-L). **(F)** Schematic representation of the two isoforms of peroxiredoxin-5 (Prdx-5) in *H. sapiens* (green) and *D. rerio* (orange). The long variant harbours an additional mitochondrial targeting signal, whereas the C-terminal sequence is identical. **(G,H)** Sequence comparison of the C-terminal end of USP2 orthologues **(G)** and PRDX5-orthologues **(H)**. The loss of the PTS1 in Usp2-L is unique for *D. rerio*, whereas the PTS1 of human PRDX5 is found only in more ancient fish species.

Alternatively, acyl-CoA products can be hydrolysed by acyl-CoA thioesterases (ACOTs) that generate a free carboxylate that can cross the peroxisomal membrane (Houten et al., 2020). Similar to humans, *D. rerio* Acot8 contains a canonical PTS1 (SKL), whereas a homologue of *Hs*ACOT4 was not identified (Table 1 and Supplementary Table S3). Interestingly, an orthologue of bile acid-CoA:amino acid N-acyltransferase (BAAT), which in humans converts choloyl-CoA and deoxycholoyl-CoA to taurine- or glycine-conjugated cholic acid, or deoxycholic acid, was not identified in *D. rerio* (Table 1 and Supplementary Table S3). As Cypriniformes (including *D. rerio*) lack C24 bile acids (which are formed by side chain shortening in human peroxisomes), a peroxisomal contribution to bile acid synthesis in zebrafish is rather unlikely (Hagey et al., 2010). This is also supported by the absence of ACOX2, which in humans is involved in bile acid biosynthesis. Comparison with various other fish species demonstrate that BAAT is found in neither of these species (Figure 4D).

Mitochondrial β-oxidation relies on acyl-CoA dehydrogenases (ACADs). Interestingly, peroxisomes also harbour an acyl-CoA dehydrogenase, ACAD11 (Camões et al., 2015). Like the human ACAD11, the *D. rerio* Acad11 has a canonical PTS1 (AKL) (Table 1 and Supplementary Table S3). If the enzyme contributes to the 1st step of peroxisomal β-oxidation is not known; however, this could circumvent generation of H_2_O_2_ by peroxisomal ACOX (Camões et al., 2015). A potential Acad11 isoform was also identified, which only contains the N-terminal aminoglycoside phosphotransferase (APH) domain (E9QBY4_DANRE) but lacks a PTS1. Fungi still contain independent enzymes with the APH and ACAD function, which appear to be fused during evolution. However, in fungi both individual enzymes possess a PTS1 (Camões et al., 2015).

Whereas a mitochondrial fatty acid β-oxidation pathway is absent in yeast and plants, which solely rely on the peroxisomal pathway, it is present in animals and many other fungi. Zebrafish also encodes a set of homologous enzymes of human mitochondrial β-oxidation, e.g., short-chain to very long-chain specific acyl-CoA dehydrogenases (Acadvl, F1Q8J4_DANRE; Acadl, A3KQR0_DANRE, Acadm, A2CG95_DANRE; Acads Q6AXI7_DANRE) for the initial dehydrogenation reaction of fatty acids, and enoyl-CoA hydratases (Hadhaa, A7YT47_DANRE; Echs1, Q7ZZ04_DANRE) for the 2nd step. Zebrafish candidates for the third and fourth reaction of mitochondrial β-oxidation are again Hadhaa or 3-hydroxyacyl-CoA dehydrogenase (Hadh, Q6DI22_DANRE) and the 3-ketoacyl-CoA thiolases (Hadhb, A0A2R8RRC9_DANRE; Acat1, Q6AZA0_DANRE) and acetyl-CoA acyltransferase 2 (Acaa2, B0S5C5_DANRE). Hence, fatty acid substrate spectra metabolized in peroxisomes or mitochondria seem to closely resemble the situation in human. This is also corroborated by the specific increase of very long chain fatty acids in zebrafish knockout models for peroxisomal disorders (Takashima et al., 2021).

### Peroxisomal Fatty Acid Alpha-Oxidation

Our comprehensive analysis revealed candidate proteins for several other important metabolic pathways in *D. rerio* peroxisomes (Table 1). Due to a methyl group at position 3, 3-methyl branched-chain fatty acids such as phytanic acid cannot undergo β-oxidation directly. Peroxisomal α-oxidation is required to remove the last carbon atom generating a 2-methyl branched-chain fatty acid, which can be β-oxidised (Wanders and Waterham, 2006). *D. rerio* possesses a phytanoyl-CoA 2-hydroxylase (Phyh) carrying a PTS2 for hydroxylation of phytanoyl-CoA (Table 1 and Supplementary Table S3). The generated 2-hydroxyphytanoyl-CoA is cleaved by 2-hydroxyacyl-CoA lyase (Hacl1) containing a PTS1 (SNL) to pristanal and formyl-CoA. Pristanal is oxidised to pristanic acid by a peroxisomal aldehyde dehydrogenase, which in humans is FALDH, a tail-anchored membrane protein with a peroxisomal and an ER-targeted isoform (Costello et al., 2017b). *D. rerio* possesses two homologous fatty aldehyde dehydrogenases (Aldh3a2a, Aldh3a2b), which are also tail-anchored membrane proteins (Table 1 and Supplementary Table S3). However, their peroxisomal or ER localisation needs to be determined.

### Biosynthesis of Ether Phospholipids

The biosynthesis of ether phospholipids (e.g., myelin sheath lipids and plasmalogens) is a characteristic feature of peroxisomes in animals, but supposed to be absent in plants and yeast (Dorninger et al., 2020). It requires metabolic cooperation of peroxisomes with the ER. The formation of the typical ether bond in ether phospholipids is catalysed by the peroxisomal enzyme alkyl-dihydroxyacetone phosphate synthase (alkyl-DHAP synthase) (Figure 3B). In *D. rerio*, this key enzyme (Agps) possesses a PTS2 (Table 1 and Supplementary Table S3). Formation of the two substrates for alkyl-DHAP synthase, namely acyl-DHAP and a long chain alcohol, is catalysed by DHAP acyltransferase (DHAPAT) (*D. rerio* Gnpat) carrying a PTS1 (ARL), and FAR1 or FAR2, two different acyl-CoA reductases (Table 1 and Supplementary Table S3). Like human FAR1, *D. rerio* Far1 (A0A0R4ICF6_DANRE) appears to be a tail-anchored membrane protein; however, a potential Far1 isoform without a TMD and a PTS1 (SRL) may exist (F1QSU9_DANRE) (Supplementary Table S3). An orthologue of human FAR2 was also identified (si:dkey-97m3.1, Q1L8Q4_DANRE), which appears to be a tail-anchored membrane protein as well (Table 1 and Supplementary Table S3). The DHAP-backbone (precursor compound) is reduced by the enzyme acyl/alkyl-DHAP reductase (AADHAPR) encoded by *DHRS7B* (dehydrogenase/reductase SDR family member 7B) at the cytosolic face of the peroxisomal or ER membrane. The *D. rerio* Dhrs7b possesses an N-terminal TMD (Table 1 and Supplementary Table S3).

### Glycolate/Glyoxylate Metabolism and Detoxification

Furthermore, several enzymes involved in glycolate/glyoxylate metabolism and detoxification have been identified including hydroxyacid oxidase 1 and 2 (Hao1, Hao2) with a PTS1 (Tables 1, 2 and Supplementary Table S3). Humans express three 2-hydroxyacid oxidases (HAOX1-3) in a tissue-specific manner; an orthologue of HAOX3, which may have evolved from Hao2, was not found in zebrafish. These flavin-linked enzymes convert 2-hydroxy acids to 2-keto acids thereby reducing molecular oxygen to hydrogen peroxide. HAOX1 shows a preference for the two-carbon substrate, glycolate, but is also active on 2-hydroxy fatty acids, whereas HAOX2 displays a preference for 2-hydroxypalmitate, and HAOX3 for the medium chain substrate 2-hydroxyoctanoate. These findings suggest that HAOX1-3 are involved in the oxidation of 2-hydroxy fatty acids and may also contribute to fatty acid α-oxidation (Jones et al., 2000).

**TABLE 2:** Candidate peroxisomal oxidases in zebrafish.

Name	Protein symbol	Uniprot ID	ROS/NOS	PTS1	Localisation (predicted)
Acyl-CoA oxidase 1	Acox1	F1R071_DANRE	H_2_O_2_	SKL	PO
Acyl-CoA oxidase 3	Acox3	F1QXK3_DANRE	H_2_O_2_	AKL	PO
Acyl-CoA oxidase-like	Acoxl	F1R4J4_DANRE	H_2_O_2_	–	PO?
L-α-Hydoxyacid oxidase 1	Hao1	Q7SXE5_DANRE	H_2_O_2_	SRI	PO
L-α-Hydoxyacid oxidase 2	Hao2	F1QCD8_DANRE	H_2_O_2_	SRL	PO
D-amino-acid oxidase 1	Dao1	Q4V981_DANRE	H_2_O_2_	SRL	PO
D-amino-acid oxidase 2	Dao2	Q6NY97_DANRE	H_2_O_2_	SRL	PO
D-amino-acid oxidase 3	Dao3	Q6P009_DANRE	H_2_O_2_	SRL	PO
D-aspartate oxidase	Ddo	A0A0R4IZE6_DANRE	H_2_O_2_	ARL	PO
L-Pipecolic acid oxidase	Pipox	A7MBQ2_DANRE	H_2_O_2_	SSL	PO
Polyamine oxidase	Paox	B8JJQ4_DANRE	H_2_O_2_	SKL	PO
Urate oxidase	Uox	Q6DG85_DANRE	H_2_O_2_	ARM	PO
Xanthine oxidase	Xdh	A0A2R8Q1T4_DANRE	H_2_O_2_, NO^•^, O_2^•–^_	–	Cyt
L-gulonolactone oxidase (ascorbate synthesis)	Gulo	–	H_2_O_2_	–	absent

*Inducible nitric oxide synthase (NOS2) (NO^•^, O_2^•–^_) not listed (no PTS, Cyt, PO?).*

Zebrafish also contains a putative peroxisomal alanine:glyoxylate aminotransferase (AGT) (Agxta, F1QY24 _DANRE) with a weak PTS1 (SRV), a key enzyme to prevent oxalate accumulation (Table 1 and Supplementary Table S3). The peroxisomal AGT converts glyoxylate generated in peroxisomes (by HAOX1/glycolate oxidase) into glycine using alanine as the primary amino group donor (Wanders and Waterham, 2006). This prevents the conversion of glyoxylate into oxalate, which is toxic. AGT enzymatic activities associated with mitochondria are also required to utilize certain amino acids for gluconeogenesis. Interestingly, zebrafish encode another putative AGT (agxtb, Q6PHK4_DANRE) which lacks a PTS1 but possesses an N-terminal MTS. Mitochondrial AGT can transaminate alanine and serine to pyruvate and hydroxypyruvate for gluconeogenesis. The peroxisomal and/or mitochondrial localisation of AGT in animals reflects the organism’s diet. Glyoxylate, as a metabolite of glycolate, is taken up by the consumption of plants, whereas protein-rich diets from animal sources deliver high quantities of amino acids such as glycine, alanine and serine. Therefore, AGT localises to mitochondria in many carnivores, to peroxisomes in herbivores, and to both organelles in omnivores (Danpure, 1997). In contrast to zebrafish, mammals contain only a single AGT gene, and dual localisation of AGT, e.g., in rat or marmoset is often mediated by alternative transcription/translation generating a PTS1 or MTS. In humans, AGT is exclusively peroxisomal; AGT deficiency results in primary hyperoxaluria, with accumulation and precipitation of oxalate in the liver and kidney, ultimately leading to kidney failure (Dindo et al., 2019). Hyperoxaluria type 1 can also be caused by a polymorphism (Pro11Leu) in the AGT gene in combination with the mutation Gly170Arg, which creates a MTS mislocalising the enzyme to mitochondria in homozygote patients (Leiper and Danpure, 1997). Furthermore, we identified a malate synthase-like protein (Mls1) with a PTS1 (ARL) in *D. rerio*, which is absent in *H. sapiens* (Table 1 and Supplementary Figure S3A). The enzyme can convert glyoxylate and acetyl-CoA into malate.

### Catabolism of D- and L-Amino Acids

Like mammals, zebrafish peroxisomes also appear to contain several enzymes involved in the catabolism of D- and L-amino acids. These include three putative D-amino acid oxidases (dao1-3) carrying a SRL, which oxidize the D-isomers of neutral and basic amino acids, as well as D-aspartate oxidase (Ddo), which oxidizes the D-isomers of acidic amino acids such as D-aspartate, D-glutamate, and N-methyl-D-aspartate that have important neuroregulatory functions in the central nervous system (Tables 1, 2 and Supplementary Table S3). Those enzymes generate the corresponding keto-acids, ammonia, and hydrogen peroxide (Wanders and Waterham, 2006). We also identified enzymes, which may be involved in the degradation of L-amino acids, e.g., leucine (Hmgcl, F1QTF0_DANRE) (Table 1 and Supplementary Table S3). For human peroxisomal hydroxymethylglutaryl-CoA lyase (HMGCL), a role in the regulation of ketone body metabolism has been suggested (Arnedo et al., 2019). Furthermore, a *D. rerio* L-pipecolate oxidase (Pipox) with a weak PTS1 (SSL) was identified, which likely oxidizes L-pipecolate to Δ1-piperideine-6-carboxylate (Wanders and Waterham, 2006; Tables 1, 2 and Supplementary Table S3). The enzyme is present in human and primate peroxisomes but is mitochondrial in rabbit liver.

### Polyamine Oxidation

Zebrafish also contain a candidate polyamine oxidase (Paox) with a canonical PTS1 (SKL), which in humans is involved in the degradation of spermine and spermidine (Wanders and Waterham, 2006; Tables 1, 2 and Supplementary Table S3). These natural polyamines are present in all eukaryotic cells and support essential functions in cell proliferation, differentiation and immune regulation, which requires tight control of their levels.

### Purine Catabolism

Peroxisomes also harbour enzymes for purine catabolism. In fish, amphibians and many invertebrates purine degradation is catalysed by xanthine oxidase, urate oxidase, allantoinase, and allantoicase, which produce the metabolites uric acid, allantoin, allantoic acid and urea as well as ureidoglycolate (Islinger et al., 2010) (see overview Figure 3C). In freshwater fish such as zebrafish xanthine oxidase and allantoinase are cytosolic enzymes, whereas urate oxidase (Uox, Q6DG85_DANRE) and allantoicase (Allc, Q6DGA6) are peroxisomal containing a PTS1 (Tables 1, 2 and Supplementary Table S3; Hayashi et al., 1989). The structure and activity of zebrafish urate oxidase was recently reported (Marchetti et al., 2016). Most mammals excrete allantoin and do not express allantoinase and allantoicase (Figure 3C). Furthermore, humans (as well as other primates, birds and reptiles) do not possess a functional uricase gene, thus excreting uric acid (Hayashi et al., 2000; Islinger et al., 2020). This makes humans susceptible to gout or urate kidney stones.

Other enzymes associated with urate degradation have recently been identified (Ramazzina et al., 2006; Figure 3C). In contrast to humans, zebrafish also possess a 5-hydroxyisourate hydrolase (Uraha) involved in the degradation of uric acid to (S)-allantoin (Table 1 and Supplementary Table S3). Following urate oxidation to 5-hydroxyisourate (HIU), which is catalysed by urate oxidase, hydrolysis of HIU to 2-oxo-4-hydroxy-4-carboxy-5-ureidoimidazoline (OHCU) and subsequent decarboxylation of OHCU to (S)-allantoin are catalysed by HIUase and OHCU decarboxylase (Urad), respectively (Table 1 and Supplementary Table S3). The structure of the *D. rerio* HIUase and OHCU decarboxylase has been revealed (Zanotti et al., 2006; Cendron et al., 2007). *D. rerio* Uraha contains a putative PTS2, which is also found in the amphibian or mammalian proteins (Ramazzina et al., 2006), whereas *D. rerio* Urad possesses a predicted weak PTS1 (TKL). Interestingly, inactivation by pseudogenization of the uricase gene (see above) and the HIUase and OHCU decarboxylase genes of the pathway occurred during hominoid evolution.

To verify the functionality of these targeting signals, we generated EGFP reporter proteins with the respective PTS2 or PTS1 sequence (PTS2-*Dr*Uraha; PTS1-*Dr*Urad). Expression in COS-7 cells revealed a predominantly cytoplasmic localisation of PTS1-*Dr*Urad; however, peroxisomes were also labelled confirming that the PTS1 (TKL) is weak but functional (Supplementary Figure S2). Surprisingly, PTS2-*Dr*Uraha was not targeted to peroxisomes suggesting that the PTS2 may not be functional in zebrafish (Supplementary Figure S2).

### Oxygen and ROS Metabolism

Peroxisomes harbour a number of oxidases that reduce oxygen to hydrogen peroxide (Table 2), thus contributing to oxygen and ROS metabolism (Antonenkov et al., 2010). The H_2_O_2_ produced can be decomposed by several peroxisomal enzymes. The key enzyme is catalase, a heme-binding tetrameric enzyme, which can perform a catalytic (2H_2_O_2_ → O_2_ + 2H_2_O) or peroxidatic reaction (H_2_O_2_ + AH_2_ → A + 2H_2_O), in which the conversion of H_2_O_2_ to H_2_O is coupled to the oxidation of a hydrogen donor (AH_2_) (e.g., ethanol, methanol, formaldehyde, formate, nitrite). Like other organisms, *D. rerio* catalase (Cat) contains a non-canonical, less effective PTS1 (SKM), which is supposed to allow proper folding of the protein prior to peroxisomal import (Williams et al., 2012; Table 1 and Supplementary Table S3). The peroxidatic reaction of catalase is exploited for diaminobenzidine (DAB) cytochemistry to specifically label peroxisomes for light- and electron microscopy (Fahimi, 2009), and has been successfully applied to identify peroxisomes in zebrafish tissues (Braunbeck et al., 1990; Krysko et al., 2010).

Xanthine oxidase, which generates superoxide anions, is likely not peroxisomal in zebrafish, and localises to the cytosol instead (Table 2). Superoxide radicals can be inactivated by superoxide dismutases. The presence of Cu/Zn-SOD and Mn-SOD activities in peroxisomes from mammals has been reported (Schrader and Fahimi, 2006). Cu/Zn-SOD1, which does not possess a PTS signal, is imported piggy-back into peroxisomes by its copper chaperone which carries a PTS1 (Islinger et al., 2009), but also localises to the cytosol, nucleus and mitochondria. A candidate copper chaperone for SOD is also present in zebrafish (Ccs, A0A0R4IHZ8_DANRE) showing a PTS1 (SHL), as well as superoxide dismutase (Sod1, O73872-SODC_DANRE) lacking a PTS1 (Table 1 and Supplementary Table S3). By contrast, Mn-SOD (SOD2) has recently been reported to exclusively localize to mitochondria in mice and men (Karnati et al., 2013).

Peroxisomes also contain nitric oxide (NO) synthase activity. Zebrafish NO synthases (Nos2a, Nos2b) do not possess a PTS1, however, the molecular mechanism of NO synthase targeting is currently unclear, and the enzyme may present a source of superoxide radicals in peroxisomes (Fransen et al., 2012; Table 1 and Supplementary Table S3).

Peroxiredoxins are a family of antioxidant proteins ubiquitously conserved in a wide variety of organisms ranging from bacteria to humans. In zebrafish, six peroxiredoxins (Prdx1-6) have been identified. Prdx1 is a highly abundant cytosolic thioredoxin-dependent peroxidase and appears to be more efficient at removing H_2_O_2_ and organic hydroperoxides. Prdx1 is important for vascular development in zebrafish (Huang et al., 2017), and has a stimulatory role in the initiation of adaptive humoral immunity (Liu et al., 2018). Different isoforms of human peroxiredoxin-5 exist which localise to the cytosol, mitochondria or peroxisomes. The peroxisomal isoform possesses a PTS1 (Yamashita et al., 1999), whereas the *D. rerio* Prdx5 (Prdx5, F1QCE3_DANRE) lacks a PTS1 or PTS2 (Figure 4, Table 1, and Supplementary Table S3). Thus, neither of the *D. rerio* Prdx1-6 appears to possess a PTS1.

Peroxisomes, in addition to mitochondria, also contain glutathione S-transferase kappa (GSTK1), which is supposed to catalyse the conjunction of xenobiotics and lipid peroxide products with glutathione for detoxification (Fransen et al., 2012). The peroxisomal localisation of GSTK1 depends on a PTS1 (Morel et al., 2004), which is also present in *D. rerio* Gstk1 (PTS1 AKM) (Table 1 and Supplementary Table S3).

Finally, a candidate for *D. rerio* epoxide hydrolase 2 (Ephx2) with a PTS1 has been identified (Table 1 and Supplementary Table S3). In mammals, EPHX2 localises to the cytosol and peroxisomes carrying a weak PTS1 (Mullen et al., 1999). The homodimeric enzyme is supposed to detoxify fatty-acid derived epoxides converting them to the corresponding dihydrothiols. Downregulation or inhibition of epoxide hydrolase in zebrafish impaired the development of the caudal vein plexus (Frömel et al., 2012).

### Peroxisomal Proteases

We also identified *D. rerio* candidate proteins for the three peroxisomal proteases identified so far. Whereas insulin-degrading enzyme (Ide) may degrade oxidised proteins in peroxisomes, Lon protease homolog 2 (Lonp2) is an ATP-dependent protease that mediates the selective degradation of matrix proteins damaged by oxidation (Bartoszewska et al., 2012) as well as sorting and processing of PTS1-containing proteins (Omi et al., 2008). Trypsin domain-containing 1 (Tysnd1) processes several PTS1-containing proteins, cleaves N-terminal presequences from PTS2-containing protein precursors, and proteolytic processing of β-oxidation enzymes (Kurochkin et al., 2007; Okumoto et al., 2011). All three *D. rerio* proteases possess a PTS1 (Table 1 and Supplementary Table S3).

### Carbohydrate Metabolism

Peroxisomes also contain enzymes involved in carbohydrate metabolism, for example the two pentose phosphate pathway enzymes, glucose-6-phosphate dehydrogenase and 6-phosphogluconate dehydrogenase (Wanders and Waterham, 2006). It is supposed that these enzymes provide intraperoxisomal NADPH from NADP^+^, which is generated during beta-oxidation of unsaturated fatty acids by 2,4- dienoyl-CoA reductase (DECR2) and through conversion of phytenoyl-CoA into phytanoyl-CoA by trans-2-enoyl-CoA reductase (PECR). Whereas the candidate *D. rerio* glucose 6-phosphate dehydrogenase (G6pd) possesses a weak PTS1 (HKL), the candidate 6-phosphogluconate dehydrogenases (Pgd) do not possess a PTS1 (Table 1 and Supplementary Table S3). Alternatively, intraperoxisomal NADPH can be regenerated by isocitrate dehydrogenase 1 (IDH1) in a peroxisomal 2-oxoglutarate/isocitrate NADP(H) redox shuttle (reviewed in Chornyi et al., 2020). The *D. rerio* Idh1 contains a PTS1 (PKL) indicating that this shuttle system also exists in zebrafish (Table 1 and Supplementary Table S3).

NADH, which is produced from NAD^+^ during peroxisomal α- and β-oxidation, may be reoxidised to NAD^+^ by different NAD^+^-dependent dehydrogenases, which have been reported to reside in human peroxisomes and may contribute to a NAD^+^/NADH shuttle system: (i) lactate dehydrogenase B (LDHB), which converts pyruvate into lactate, (ii) malate dehydrogenase 1 (MDH1), which converts oxaloacetate into malate, and (iii) glycerol-3-phosphate dehydrogenase 1 (GPD1), which converts DHAP into G3P (reviewed in Chornyi et al., 2020). Human LDHA and LDHB both lack PTS signals, but for LDHB a translational read-through of the stop codon was reported to result in an alternative, C-terminally extended isoform with a PTS1 (Schueren et al., 2014). Similarly, MDH1 does not possess a PTS, but translational read-through to generate an extended isoform with a functional PTS1 has been reported (Hofhuis et al., 2016). Interestingly, an isoform of malate dehydrogenase 1Aa (Mdh1x, NP_001303854.1) carrying a PTS1 (SRL) was identified in zebrafish, which is generated by translational read through of the UGA stop codon to the downstream UAA termination codon generating a C-terminally extended isoform (Stiebler et al., 2014; Table 1 and Supplementary Table S3). Whereas a Gpd1-based redox shuttle has been described in yeast (with *Sc*Gpd1 carrying a PTS2), it is unclear how mammalian Gpd1 targets peroxisomes as it lacks a PTS and does not show translational read-through (reviewed in Chornyi et al., 2020). *D. rerio* Gpd1 (Q567A1_DANRE; Q5XIZ6| GPD1L_DANRE) does not appear to possess a PTS2 or PTS1 signal. The same applies to the *D. rerio* lactate dehydrogenases (Ldhb), and it appears that translational readthrough and peroxisomal targeting of Ldhb is restricted to mammals (Hofhuis et al., 2016). Furthermore, the higher degree of conservation of MDH1 readthrough in comparison to LDHB readthrough co-evolved with the targeting signal strength of their respective PTS1. Thus, there is evidence for a peroxisomal oxaloacetate/malate NAD^+^/NADH shuttle system in zebrafish, whereas the peroxisomal localisation of other NAD^+^-dependent dehydrogenases and their role in a NAD^+^/NADH shuttle system need to be elucidated.

### Peroxisomal CoA Transporter

The import and export of small metabolites (such as pyruvate, lactate, oxaloacetate, malate, DHAP, G3P, isocitrate, and 2-oxoglutarate) is likely mediated by peroxisomal pore-forming proteins and transporters. *D. rerio* possesses two orthologues of the human peroxisomal transmembrane protein PMP34/SLC25A17 (Slc25a17, A5D6T2_DANRE; Slc25a17-like), which in zebrafish resulted from gene duplication and function as a peroxisomal CoA transporter (Kim et al., 2020; Figure 2, Table 1, and Supplementary Table S3). These members of the solute carrier protein family (SLC) exhibit six transmembrane domains, and have been shown to localise to peroxisomes in zebrafish embryos (Kim et al., 2020). Targeting of PMPs is mediated by the PMP import receptor/chaperone PEX19. Knockdown of Slc25a17 impaired development of multiple organs, including swim bladder, during zebrafish embryogenesis. Furthermore, the concentration of VLCFA was increased after Slc25a17 knockdown, whereas the concentration of plasmalogens was decreased supporting a role in lipid metabolism/cofactor transport (Kim et al., 2020).

CoA is an important co-factor in peroxisomal lipid metabolism, which is released during hydrolysis of acyl-CoA esters by the thioesterases (*see* section “Fatty acid β-oxidation”). It is re-used inside peroxisomes (e.g., for the activation of pristanic acid), or is degraded by one of the peroxisomal Nudix Hydrolases (NUDT). Orthologues of the human Nudix Hydrolases NUDT7 and NUDT19/RP2p, two CoA diphosphohydrolases that degrade CoA and acyl-CoAs to 3′,5′-ADP and 4′-(acyl)phosphopantetheine, are also present in zebrafish. *D. rerio* Nudt7 and Nudt19 have a canonical PTS1 (SKL), but the Nudt19 PTS1 signal appears to be weak and may allow targeting to other subcellular compartments (Table 1 and Supplementary Table S3). In addition, the pyrophosphatase NUDT12, which mediates the degradation of NAD^+^ and NADH, was reported to target human peroxisomes. However, the *D. rerio* Nudt12 does not possess a predicted PTS1 and may localise to the cytosol.

### Peroxisomal Membrane Proteins

Orthologues of the human peroxisomal membrane proteins PXMP2 (PMP22), PXMP4 (PMP24) and PMP52 (TMEM135) were also identified in *D. rerio* (Pxmp2, Pxmp4, Tmem135) (Figure 2, Table 1, and Supplementary Table S3). PXMP2 is supposed to be a pore-forming protein; PXMP4 and TMEM135 belong to the Tim17 family and they may be involved in peroxisomal metabolite transport (reviewed in Chornyi et al., 2020; Beasley et al., 2021). Furthermore, orthologues of human membrane proteins MPV17 and MPV17-like are present in zebrafish (Mpv17, Mpv17l, Mpv17l2), which belong to the Mpv/Pxmp2 family (Figure 2, Table 1, and Supplementary Table S3). Their peroxisomal localisation is controversial (Iida et al., 2006; Spinazzola et al., 2006; Weiher et al., 2016), and Mpv1 appears to be an inner mitochondrial membrane protein (Martorano et al., 2019). It possesses a weak predicted PTS1 (NKM), but as a multi-pass membrane protein, peroxisomal targeting would depend on Pex19. The two *D. rerio* Mpv17-like proteins are as well multi-pass membrane proteins.

Furthermore, only one orthologue of the human peroxisomal membrane proteins MARC1/2 is present in *D. rerio* (Marc1, Q66HU7_DANRE) (Figure 2, Table 1, and Supplementary Table S3), which is most likely due to a recent gene duplication in amniota after the separation from fish. MARC2, which has been identified in proteomics studies of mammalian peroxisomes, mediates the reduction of N-hydroxylated drugs in mitochondria (Havemeyer et al., 2006), but its peroxisomal function is unknown. Interestingly, MARC2 knockout mice exhibit lower body weight and are resistant to high-fat-diet induced obesity linking MARC2 function to the regulation of energy homeostasis (Rixen et al., 2019). If *D. rerio* Marc localises to peroxisomes needs to be determined. Other putative peroxisomal membrane proteins identified in *D. rerio* are the mitochondrial antiviral-signaling protein Mavs, a tail-anchored membrane protein, which in humans localises to mitochondria and peroxisomes (Dixit et al., 2010); the AAA-ATPase Msp1/Atad1, which dually localises to mitochondria and peroxisomes and is involved in the quality control of tail-anchored membrane proteins (Jiang, 2021); the deubiquitinase Usp30, which is regulating the turnover of peroxisomes by suppressing basal pexophagy (Marcassa et al., 2019; Riccio et al., 2019); the putative organic cation transporter Slc22a21, which may be involved in carnitine transport (reviewed in Chornyi et al., 2020), Trim37, an E3 ubiquitin-protein ligase involved in Pex5 mediated peroxisomal matrix protein import (Wang et al., 2017), and the phospholipase Pnpla8 (Figure 2, Table 1, and Supplementary Table S3). The latter has been show to localise to mitochondria and peroxisomes, where it may be involved in the maintenance of the organelle’s membrane phospholipids, and possesses a canonical PTS1 (SKL) (Mancuso et al., 2004, 2007). Pnpla8 was suggested to peripherally associate to the inner leaflet of the peroxisome membrane after import into the peroxisomal matrix (Mancuso et al., 2000). Furthermore, Fibronectin type III domain containing 5, a putative single-pass transmembrane protein, containing a PTS1 (SKV) has been identified (G1K2P4_DANRE) (Table 1 and Supplementary Table S3). Mouse Fndc5/PeP has been shown to target peroxisomes via its PTS1 in a Pex5-dependent manner (Ferrer-Martínez et al., 2002). Murine Fndc5 is also supposed to target the plasma membrane and contains an N-terminal signal peptide. Interestingly, N-terminally truncated isoforms lacking the signal peptide exist in zebrafish (G1K2P4_DANRE) and humans (Q8NAU1-4). If those isoforms target peroxisomes via their PTS1/PEX5 or mPTS/PEX19 remains to be elucidated.

L-gulonolactone oxidase, a single pass membrane protein, is the ultimate enzyme of hepatic ascorbate formation in ascorbate-synthesizing species. In mouse liver, it localises to the ER and peroxisomes (Braun et al., 1999). However, the enzyme is absent in teleost fish and also humans, who do not synthesize ascorbate (Fracalossi et al., 2001; Tables 1, 2 and Supplementary Table S3).

### Analysis of Peroxisomal Targeting Signals

After annotation of the peroxisomal protein inventory of *D. rerio*, we analysed the peroxisomal targeting signals in more detail. We revealed that similar to other vertebrates/animals, the majority of the peroxisomal matrix proteins of *D. rerio* contain a PTS1, and only a few possess a PTS2 (Table 1 and Supplementary Table S3). The latter include peroxisomal 3-ketoacyl-CoA thiolase (Acaa1), a protein exerting the last step of peroxisomal fatty acid β-oxidation (see above), phytanoyl-CoA 2-hydroxylase (Phyh), a key enzyme of peroxisomal fatty acid α-oxidation, and alkylglycerone-phosphate synthase (Agps), a key enzyme of peroxisomal ether phospholipid biosynthesis (Table 1 and Supplementary Table S3). The human orthologues of these enzymes also possess a PTS2. Furthermore, urate (5-hydroxyiso-) hydrolase a (Uraha), an enzyme involved in urate catabolism, which is not expressed in humans, carries a putative PTS2 (Ramazzina et al., 2006), but may not target peroxisomes in zebrafish (Supplementary Figure S2). The zebrafish orthologues of other human proteins with a putative PTS2 (Kv channel-interacting protein 4 isoform 1; von Willebrand factor A domain-containing protein 8) do not possess a predicted PTS2.

To verify that the characteristic pattern of PTS1 motifs was conserved between fish and humans, we compiled the known human PTS1-carrying matrix proteins and their *D. rerio* orthologues and plotted the relative abundance of amino acids at each position of the PTS1 (Figures 4A,B). The patterns were very similar, and a direct comparison using SeqLogo^6^ did also not suggest any substantial differences (Supplementary Figure S3B). To visualize the differences in the PTS1 motifs of all proteins included in this study, we plotted either the prediction of fish and human PTS1 sequences as x,y-plot (Figure 4C) to show differences on the population scale or compared them side-by-side (Supplementary Figure S3A). We found that in a number of proteins the PTS1 estimation changed from “targeted” to “twilight,” reflecting a putative reduction in efficiency, whereas in several proteins a PTS1 was gained or lost. To investigate the loss of PTS1 signals in selected *D. rerio* proteins further, we also included other fish species in our analysis (Figure 4 and Supplementary Figure S3C).

Overall, our analysis revealed that two well-known mammalian peroxisomal proteins, bile acid-CoA:amino acid N-acyltransferase (BAAT) and zinc binding alcohol dehydrogenase domain containing 2/prostaglandin reductase 3 (ZADH2/PTGR3) are absent in *D. rerio* (Table 1). A previous study revealed that mouse PTGR3 is a novel 15-oxoprostaglandin-Δ(13)-reductase with a critical role in the modulation of adipocyte differentiation through the regulation of PPARγ activity (Yu et al., 2013). Whereas BAAT appears to be absent in fish, ZADH2 is present in other fish species and possesses (with the exception of Siluriformes, a sister group of Cypriniformes) a conserved PTS1 (SKL), which is also present in the human protein (Figure 4D and Supplementary Figure S3C). These findings highlight specific alterations in Cypriniform evolution with respect to peroxisomal protein inventory and metabolic functions.

Peroxiredoxin 5 (Prdx5) has lost its PTS1 in *D. rerio* and in most teleost fish species, whereas human PRDX5 possesses a PTS1, which is also retained in more ancient fish species (Figures 4D,F,H) suggesting a subcellular relocation of an originally peroxisomal protein in teleosts. Ubiquitin carboxyl-terminal hydrolase 2 (Usp2) has also lost its PTS1 in *D. rerio*. This only applies to the long isoform, Usp2-L (XP_005157570.1), which contains a PTS1 in several other fish species and in *H. sapiens* USP2 (Gousseva and Baker, 2003; Figures 4D,E,G). The short isoform, Usp2-S (Usp2b, F1RDC7_DANRE), possesses a PTS1 in *D. rerio*, *H. sapiens* (USP2-S; NP_004196.4) and in most other fish species analysed (Figures 4D,E,G).

Other *D. rerio* proteins with a negative PTS1 score include Crot, Acsl1, and Nudt12 (Figure 4C and Supplementary Figure S3A). It should however be noted that the PTS1 signal of the human orthologues, the carnitine octanoyltransferase CROT and the acyl-CoA synthetase ACSL1, is unclear and that the pyrophosphatase NUDT12 has only a weak PTS1.

Peroxisomal proteins with a PTS1 only identified in *D. rerio*, but not in *H. sapiens*, include urate oxidase (Uox), which is involved in purine catabolism (see above) (Figure 4C and Supplementary Figure S3A). Due to differences in the secretion products of purine degradation, several other enzymes of this pathway are absent in *H. sapiens*, often through pseudogenization (Figure 3C and Table 1). Furthermore, a malate synthase-like protein (Mlsl, A0A0R4IAX5_DANRE) appears to be present in *D. rerio* peroxisomes, but is absent in *H. sapiens* (Figure 4C, Table 1, Supplementary Figure S3A, and Supplementary Table S3). Malate synthase is a key enzyme of the glyoxylate cycle required for carbohydrate synthesis from acetyl-CoA, which exists in fungi, plants and bacteria. Malate synthase-like genes have, however, also been identified in genomes from most fish, amphibiens and marsupials (Kondrashov et al., 2006). In fungi and plants, malate synthase is localized in peroxisomes. Animal malate synthase-like proteins are functionally not characterized and have a patchy distribution of predicted PTS1 sequences, which we found in teleost fish and marsupials, but not in amphibians, monotremata and cartilaginous fish. In some animals, the degradation pathway of purines can lead to the generation of glyoxylate through the cleavage of allantoic acid by allantoicase activity (Kunze and Hartig, 2013). A possible function of Mlsl may thus be to condense the glyoxylate derived from purine degradation with acetyl-CoA to provide the versatile metabolite malate.

Other PTS1 containing proteins have an altered targeting prediction. Compared to the human orthologues, *D. rerio* Agt, Decr2, Hsdl2, and Nudt19 have a less efficient predicted PTS1, whereas Gstk1, Dhrs4, Hsd17b4 (Dbp), and Serhl appear to have a more efficient PTS1 signal (Table 1, Supplementary Figure S3A, and Supplementary Table S3).

In summary, our results demonstrate that although the average properties of PTS1 motifs do not differ significantly between the species (Figures 4A,B), differences are observed at the level of the individual proteins as outlined above (Figure 4C and Supplementary Figure S3A).

### Identification of Predicted Novel Peroxisomal Candidate Proteins in Zebrafish

Our prediction also contributed to the identification of potentially novel peroxisomal proteins in zebrafish and humans. Interestingly, in addition to Acot8 (Table 1 and Supplementary Table S3), we found several PTS1-containing acyl-CoA thioesterases in *D. rerio* (Acot14-18, Acot20) (Table 1 and Supplementary Tables S3, S4). ACOTs hydrolyze acyl-CoAs to the free fatty acid and CoA, and are important for the regulation of their intracellular levels. The identified Acots and their isoforms all carry a PTS1 (AKL; Acot20: SML), but may also localise to other compartments such as mitochondria, ER or the cytosol. (Table 1 and Supplementary Tables S3, S4). Several human ACOTs as well contain a potential PTS1 sequence: *Hs*ACOT2 localises to mitochondria (Hunt et al., 2006), but its isoforms possess a PTS1 (SKV), and may thus target mitochondria and peroxisomes. However, the C-terminal sequence of *Hs*ACOT2 is identical to *Hs*ACOT1, which localises to the cytosol and does not target peroxisomes (Hunt et al., 2006). Of the human ACOTs, ACOT4 and ACOT8 have been localised to peroxisomes. *Hs*ACOT6 (Q3I5F7, A0A2R8Y7H3) possesses a predicted weak PTS1 (SKI) indicating that other human ACOT family members may also localise to peroxisomes. Remarkably, mice possess a gene cluster of six ACOT genes with three (ACOT3-5) localizing to peroxisomes (Hunt et al., 2006). When aligned with the mammalian sequences, zebrafish Acot20 appears to be most closely related to the human ACOTs 1–6, while zebrafish Acot 14-18 build a more independent sequence cluster. Apparently, multiple gene duplications and subsequent losses resulted in a complex pattern of the ACOT gene family in different animals.

We also identified an orthologue of human β-Lactamase-like protein 2 (LACTB2), a zinc-binding endoribonuclease, which has recently been localised to mitochondria (Levy et al., 2016). However, the enzyme has also been identified in proteomics studies of rat and human liver peroxisomes (Islinger et al., 2007; Gronemeyer et al., 2013). The *D. rerio* Lactb2 possesses a predicted PTS1 (SNL), as do humans (Q53H82; -AHL), whereas the rat orthologue encodes a mutation (Q561R9; -ASL) (Table 1 and Supplementary Table S3). Myc-*Rn*LACTB2 did, however, not target peroxisomes when expressed in COS-7 cells, and remained in the cytosol (Camões et al., 2015). As proteins with both a mitochondrial and peroxisomal targeting signal exist (Costello et al., 2018), peroxisomal targeting of Lactb2 may depend on certain environmental conditions. Many of the proteins dually localized to peroxisomes and mitochondria are associated with fatty acid and ROS metabolism. A competitive recognition process between the peroxisomal and mitochondrial targeting machineries has been suggested. This may involve a hierarchical targeting system where individual receptor proteins scan a nascent protein in chronological order (Neuberger et al., 2004; Kunze and Berger, 2015). Examples for such a hierarchical sorting are the human alanine-glyoxylate aminotransferase (AGT), where specific mutations in the N-terminus create an MTS (see above), and L-bifunctional protein (EHHADH), where N-terminal mutations result in a dual peroxisomal and mitochondrial localisation (Klootwijk et al., 2014). However, how the dual targeting is prioritized under physiological conditions, is largely unknown (see Costello et al., 2018 for a recent review on dual targeting to peroxisomes and mitochondria).

**FIGURE 5:**
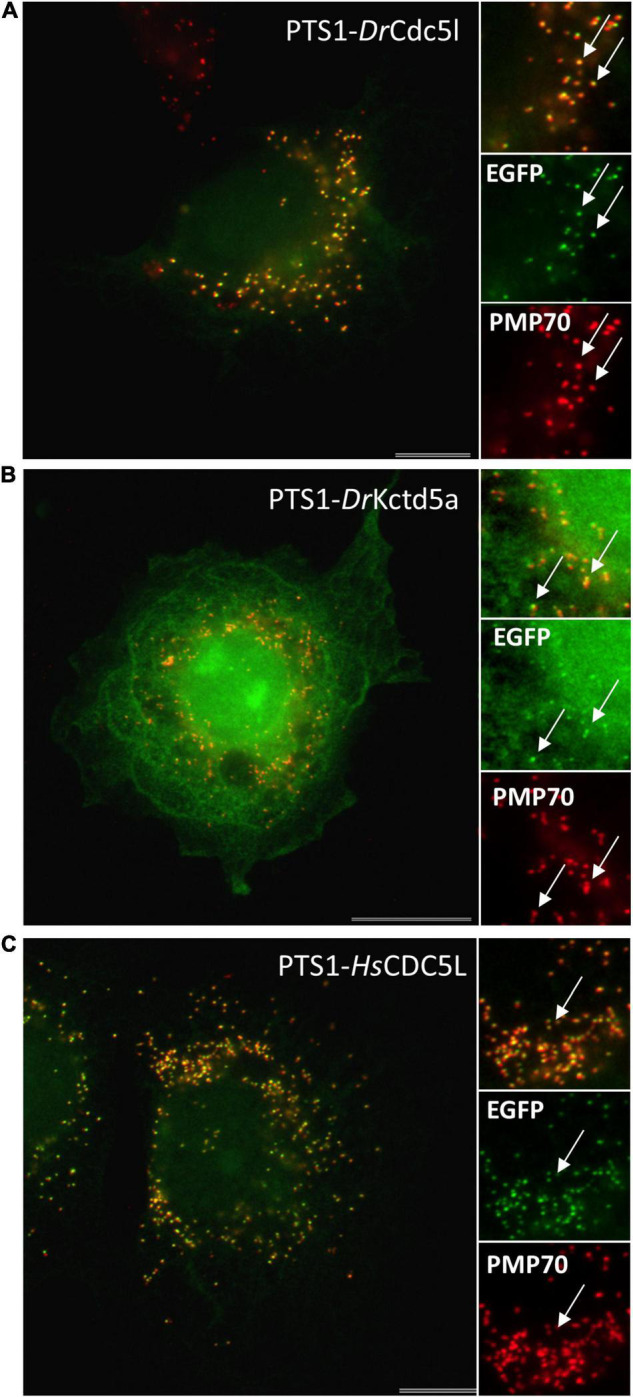
Verification of PTS1 functionality for selected candidate proteins. COS-7 cells were transfected with an EGFP fusion protein containing the C-terminal putative PTS1 of *D. rerio* Cdc5l DLLMLDKQTLSSKI **(A)**, *D. rerio* Kctd5a DLKAKILQEQGSRM **(B)**, and *H. sapiens* CDC5L DLLLEKETLKSKF **(C)**. Cells were processed for immunofluorescence microscopy using anti-PMP70 (red) as a peroxisomal marker. White arrows highlight peroxisomes that are clearly detectable in both channels. Bars, 10 μm **(A,C)**, 20 μm **(B)**.

Surprisingly, we identified several nuclear proteins with a potential PTS1 in zebrafish including the nuclear pore complex protein Nup93, anaphase-promoting complex subunit 5 (Anapc5), cell division cycle 5-like protein (Cdc5l), bromodomain adjacent to zinc finger domain 1A (Baz1a), BRISC and BRCA1-A complex member 2 (Babam2), calcium/calmodulin-dependent protein kinase I (Camk1), mesenchyme homeobox 2 (Meox2), and potassium channel tetramerization domain-containing 5 (Kctd5) (Supplementary Tables S4, S5). To verify the functionality of the PTS1 encoded in the *D. rerio* proteins Meox2a (E7FEH1), Cdc5l (E9QIC1), and Kctd5 (Q6NYY3), we generated expression plasmids for EGFP variants extended by the last 12 amino acids of these proteins. When expressed in COS-7 cells, PTS1-Meox2a was not targeted to peroxisomes (Supplementary Figure S2), whereas PTS1-Cdc5l showed a clear peroxisomal localisation (Figure 5). In addition, PTS1-Kctd5 localised to peroxisomes, but also showed a strong cytoplasmic background (Figure 5). This is consistent with the PTS1 peroxisomal targeting prediction, which indicates “targeting” for Cdc5l, but “twilight zone” for Meox2a and Kctd5 (Supplementary Tables S4, S5). However, the potential weak Meox2a and Kctd5 PTS1 appear to be conserved in different vertebrate species (Supplementary Table S5).

Putative PTS1 signals were also found in the human orthologues with predicted targeting for *Hs*CDC5L (Q99459) and *Hs*KCTD5 (Q9NXV2) (Supplementary Table S5). To verify the functionality of the human PTS1-CDC5L, which slightly differs from the *D. rerio* PTS1, we also generated an EGFP fusion. The PTS1-CDC5L was clearly targeted to peroxisomes confirming the functionality of the human PTS1 signal (Figure 5).

**FIGURE 6:**
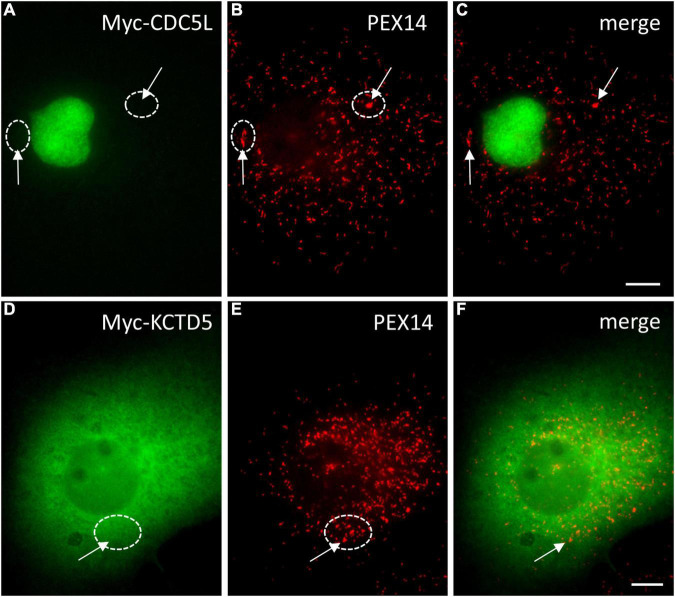
Localisation of *Hs*CDC5L and *Hs*KCTD5 in COS-7 cells. COS-7 cells were transfected with Myc-CDC5L **(A–C)** or Myc-KCTD5 **(D–F)** and processed for immunofluorescence microscopy using anti-Myc and anti-PEX14 (peroxisomal marker) antibodies. Note that a peroxisomal localisation is not detected (arrows), even at large peroxisomal structures (circles). Bars, 10 μm.

To investigate peroxisomal targeting of the human nuclear candidates with a PTS1, we selected *Hs*KCTD5 (SRM) and *Hs*CDC5L (SKF) because of their targeting sequence and accessibility of the PTS1 within the C-terminus (based on protein structure prediction). Expression of N-terminally Myc-tagged CDC5L (Myc-CDC5L) in COS-7 cells resulted in nuclear targeting, however, co-localisation with the peroxisomal marker PEX14 was not observed (Figure 6). Myc-KCTD5 localised mainly to the cytosol, but a peroxisomal localisation was also not detected (Figure 6). Our findings indicate that the PTS1 of human CDC5L (and zebrafish Cdc5L) is functional; furthermore, the PTS1 sequences of some of the nuclear candidate proteins identified appear to be maintained across different vertebrate or mammalian species. Nevertheless, full-length CDC5L is not efficiently targeted to peroxisomes in our experimental setup. An explanation may be a hierarchy of targeting signals (Neuberger et al., 2004) with a functional NLS dominating over a PTS1 under standard conditions. However, targeting may be regulated by additional factors, which may be cell type-specific or are activated under certain physiological conditions. As recently described for the nuclear transcription factor FOXM1, which translocates into mitochondria to inhibit oxidative phosphorylation (Black et al., 2020), nuclear proteins could also target peroxisomes to coordinate nuclear regulation with peroxisomal metabolism. How this may be regulated under physiological conditions is currently unknown. Our findings may inspire further studies on nuclear-peroxisome communication, a research area, which is not well explored.

## Conclusion

We reveal the first comprehensive inventory of *D. rerio* peroxisomal proteins, their targeting signals, association with peroxisomal metabolic pathways and comparison to human peroxisomes. Despite approx. 350 million years of coevolution, the protein inventories of human and zebrafish peroxisomes still appear to be largely identical implying a high degree of conservation in peroxisome metabolic functions. However, some critical changes in metabolic pathways need to be considered such as differences in purine degradation and bile acid synthesis. Our analysis confirms the suitability of zebrafish as a vertebrate model for peroxisome research and opens possibilities to study the functions of novel and established peroxisomal proteins in zebrafish in order to gain novel insights into the contribution of peroxisomes to human disorders.

## Data Availability Statement

The original contributions presented in the study are included in the article/Supplementary Material, further inquiries can be directed to the corresponding author/s.

## Author Contributions

MakK, RK, MA, MarK, and MI performed the experiments and analyzed the data. MS, MarK, MI, and VM conceived the project and analyzed the data. MS, MI, and MarK wrote the manuscript. All authors contributed to Methods.

## Conflict of Interest

MA and VM were employed by company LifeGlimmer GmbH. The remaining authors declare that the research was conducted in the absence of any commercial or financial relationships that could be construed as a potential conflict of interest.

## Publisher’s Note

All claims expressed in this article are solely those of the authors and do not necessarily represent those of their affiliated organizations, or those of the publisher, the editors and the reviewers. Any product that may be evaluated in this article, or claim that may be made by its manufacturer, is not guaranteed or endorsed by the publisher.

## Footnotes

1
www.uniprot.org

2
https://services.healthtech.dtu.dk/service.php?TMHMM-2.0

3
https://mendel.imp.ac.at/pts1/

4
https://zfin.org/

5
https://www.uniprot.org/proteomes/UP000000437

6
https://services.healthtech.dtu.dk/service.php?Seq2Logo-2.0

## Supplementary Material

Supplementary Figure S1 Alignment of PEX5 N-terminus and comparison between selected vertebrate species.

Supplementary Figure S2 Analysis of PTS1/PTS2 functionality for selected *D. rerio* candidate proteins.

Supplementary Figure S3 Differences in the PTS1-evaluation for candidate proteins.

Supplementary Table S1 Plasmids generated in this study.

Supplementary Table S2 Oligonucleotides used in this study.

Supplementary Table S3 *Danio rerio* theoretical peroxisomal protein inventory.

Supplementary Table S4 *Danio rerio* predicted novel PTS1 proteins and PTS1 conservation in humans and goldfish.

Supplementary Table S5 Selected putative *Danio rerio* nuclear proteins with a PTS1 and PTS1 conservation in vertebrate species.

## Abbreviations

PEX: peroxin
PMP: peroxisomal membrane protein
PTS: peroxisomal targeting signal
TMD: transmembrane domain
VLCFA: very long chain fatty acids.
