# Supplementary figures and images for: Insights Into the Peroxisomal Protein Inventory of Zebrafish
Source: Front Physiol. 2022 Feb 28;13:822509. doi: 10.3389/fphys.2022.822509 (PMC8919083; doi:10.3389/fphys.2022.822509)

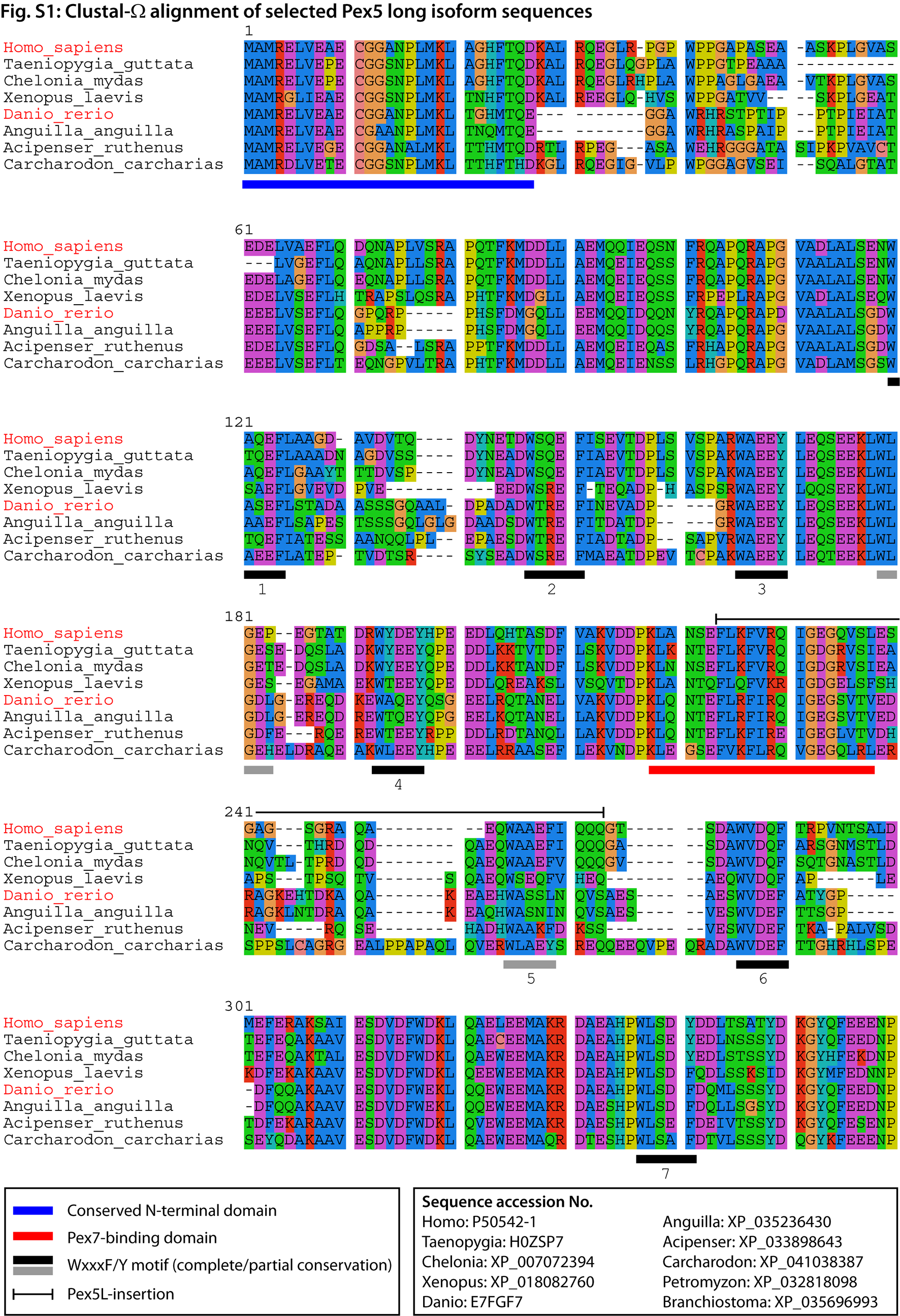

Supplement: Supplementary Figure S1 — Alignment of PEX5 N-terminus and comparison between selected vertebrate species. [file Image_1.TIF]

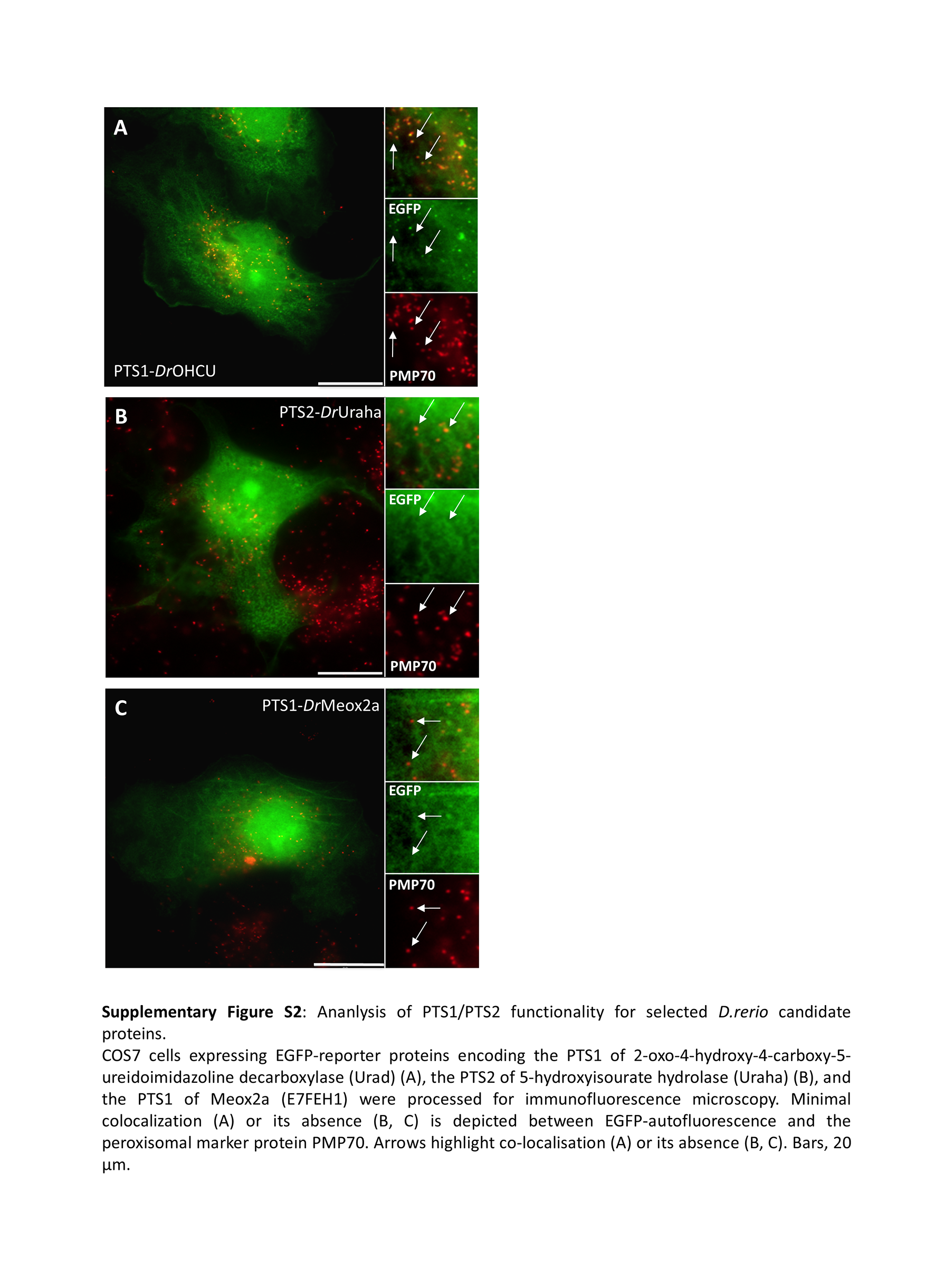

Supplement: Supplementary Figure S2 — Analysis of PTS1/PTS2 functionality for selected D. rerio candidate proteins. [file Image_2.TIF]

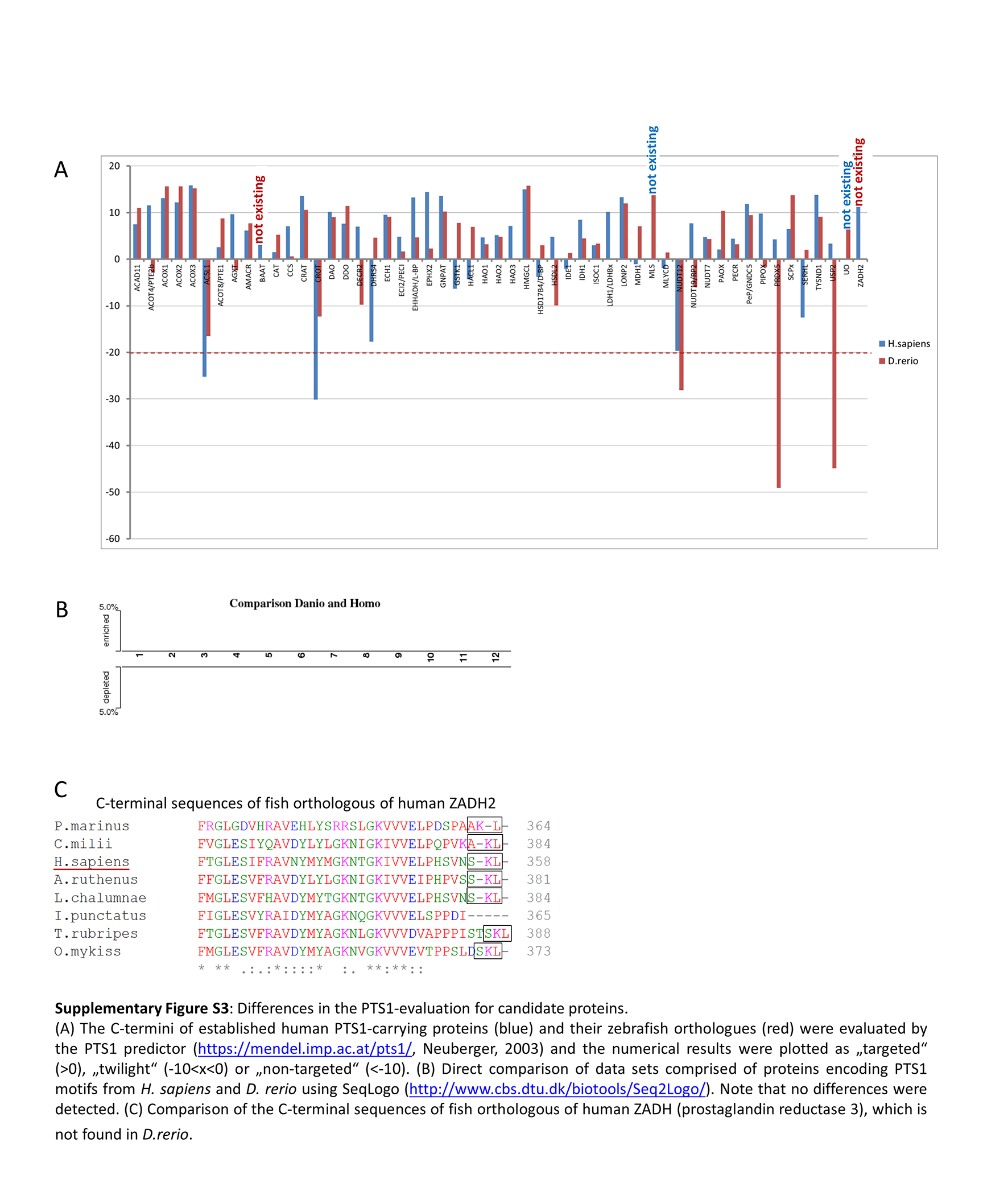

Supplement: Supplementary Figure S3 — Differences in the PTS1-evaluation for candidate proteins. [file Image_3.TIF]
