# Supplementary material for: Insights Into the Peroxisomal Protein Inventory of Zebrafish
Source: Front Physiol. 2022 Feb 28;13:822509. doi: 10.3389/fphys.2022.822509 (PMC8919083; doi:10.3389/fphys.2022.822509)
Supplement: Supplementary Table S1 — Plasmids generated in this study. [file Table_1.docx]

**Supplementary Table S1. Plasmids generated in this study**

| Plasmid | Primers | Enzymes | Vector |
| --- | --- | --- | --- |
| Myc-*Hs*CDC5L | CDC5L_Myc_Fw  CDC5L_Myc_Rv | EcoRV  SalI | pCMV-Tag3 |
| Myc-*Hs*KCTD5 | KCTD5_Myc_Fw  KCTD5_Myc_Rv | EcoRI  HindIII | pCMV-Tag3 |
| PTS2-*Dr*Urah  RLQHIRGHI | Oli_2982  Oli_2983 | EcoRI  PstI | PTS2-tester |
| PTS1-*Dr*Urad  DLHSIVLSDIQTKL | Oli_2984  Oli_2985 | BglII  HindIII | EGFP-C3 |
| PTS1-*Dr*Meox2a  DLHDSDQSSDHAHL | Oli_2986  Oli_2987 | BglII  HindIII | EGFP-C3 |
| PTS1-*Dr*Cdc5l  DLLMLDKQTLSSKI | Oli_2988  Oli_2989 | BglII  HindIII | EGFP-C3 |
| PTS1-*Dr*Kctd5a  DLKAKILQEQGSRM | Oli_2990  Oli_2991 | BglII  HindIII | EGFP-C3 |
| PTS1-HsCDC5L  DLLLEKETLKSKF | Oli_3004  Oli_3005 | BglII  HindIII | EGFP-C3 |
