# Supplementary material for: Insights Into the Peroxisomal Protein Inventory of Zebrafish
Source: Front Physiol. 2022 Feb 28;13:822509. doi: 10.3389/fphys.2022.822509 (PMC8919083; doi:10.3389/fphys.2022.822509)
Supplement: Supplementary Table S2 — Oligonucleotides used in this study. [file Table_2.docx]

**Supplementary Table S2. Oligonucleotides used in this study**

| Name | Sequence (5’ to 3’) |
| --- | --- |
| CDC5L_Myc_Fw | CACGATATCATGCCTCGAATTATGATCAAG |
| CDC5L_Myc_Rv | GTGGTCGACTCAGAATT TTGACTTTAAAGT |
| KCTD5_Myc_Fw | AACGAATTCATGGCGGAGAATCACTGCGAG |
| KCTD5_Myc_Rv | TGGAAGCTTTCACATCCTTGAGCCTCGTTC |
| Oli_2982 | GAgacttcaacatatcagaggtcatattGG |
| Oli_2983 | AATTCcAatatgacctctgatatgttgaagtctCTGCA |
| Oli_2984 | GATCTACacagtattgtcctatctgacattcaaaccaaactataaA |
| Oli_2985 | AGCTTttatagtttggtttgaatgtcagataggacaatactgtgTA |
| Oli_2986 | GATCTACacgacagcgatcagagctccgatcatgcacatttatgaA |
| Oli_2987 | AGCTTtcataaatgtgcatgatcggagctctgatcgctgtcgtGTA |
| Oli_2988 | GATCTActgatgctggacaaacagaccctcagcagcaagatctgaA |
| Oli_2989 | AGCTTtcagatcttgctgctgagggtctgtttgtccagcatcagTA |
| Oli_2990 | GATCTAAaggccaagattcttcaagaacaaggctctcgaatgtgaA |
| Oli_2991 | AGCTTtcacattcgagagccttgttcttgaagaatcttggcctTTA |
| Oli_3004 | GATCTAttgctgctggagaaagagactttaaagtcaaaattctgaA |
| Oli_3005 | AGCTTtcagaattttgactttaaagtctctttctccagcagcaaTA |
