# Supplementary material for: Insights Into the Peroxisomal Protein Inventory of Zebrafish
Source: Front Physiol. 2022 Feb 28;13:822509. doi: 10.3389/fphys.2022.822509 (PMC8919083; doi:10.3389/fphys.2022.822509)
Supplement: Supplementary Table S3 — Danio rerio theoretical peroxisomal protein inventory. [file Table_3.pdf]

Suppl. Table S3: *Danio rerio* theoretical peroxisomal protein inventory

**Accn no.**	**Name**	**Potential Function**	**PTS1**	**PTS2**	**MTS**
**Peroxins**					
ZDB-GENE-070530-1; A0A0R4IPF0_DANRE	Pex1	AAA ATPase involved in protein import into peroxisome matrix (recycling/undocking of Pex5). Localizes to cytosol and peroxisomal membrane.	n.a.	n.a.	-
ZDB-GENE-070530-2; E7F4V8_DANRE	Pex2	E(3) ligase involved in the poly-ubiquitination of Pex5. Zinc RING-finger domain.	n.a.	n.a.	-
ZDB-GENE-040426-979; Q5RIV3_DANRE	Pex3	Involved in protein import into peroxisome membrane. Docking factor for Pex19, vesicle assembly during *de novo* formation. Integral component of peroxisomal membrane.	n.a.	n.a.	●
ZDB-GENE-040426-981; E7FGF7_DANRE	Pex5	Involved in protein import into peroxisome matrix, docking. Peroxisomal matrix protein import PTS1-receptor. Localizes to cytosol and peroxisomal membrane. TPR domain.	n.a.	n.a.	-
ZDB-GENE-070705-298; F1QM21_DANRE	Pex5la	Pex5-related (PEX5R). TPR domain	n.a.	n.a.	-
ZDB-GENE-111221-1; E7F507_DANRE	Pex5lb	Pex5-related (PEX5R). TPR domain	n.a.	n.a.	-
ZDB-GENE-081104-252; F1QMB0_DANRE	Pex6	AAA ATPase involved in protein import into peroxisome matrix (recycling/undocking of Pex5). Localizes to cytosol and peroxisomal membrane.	n.a.	n.a.	●
ZDB-GENE-050320-105; A8KBW8_DANRE	Pex7	Involved in protein import into peroxisome matrix. Peroxisomal matrix protein import PTS2-receptor. Localizes to cytosol and peroxisomal membrane. WD40 domain (β-propeller).	n.a.	n.a.	-
ZDB-GENE-041010-71;	Pex10	Involved in protein import into peroxisome matrix. E(3) ligase for	n.a.	n.a.	-

Q5XJ92_DANRE		ubiquitination of Pex5. Localizes to peroxisomal membrane. Zinc RING-finger domain.			
ZDB-GENE-050419-121; A3QJY9_DANRE	Pex11a	Involved in peroxisome division/proliferation and regulation of peroxisome size. Integral component of peroxisomal membrane.	n.a.	n.a.	-
ZDB-GENE-060825-289; Q0P453_DANRE	Pex11b	Involved in peroxisomal growth and division/proliferation. Peroxisome elongation, membrane remodelling for subsequent division, activation of DRP1 GTPase activity. Integral component of peroxisomal membrane.	n.a.	n.a.	-
ZDB-GENE-050913-79; Q4V8Z0_DANRE	Pex11g	Involved in peroxisomal growth and division/proliferation. Peroxisome elongation, membrane bending for subsequent division. Integral component of peroxisomal membrane.	n.a.	n.a.	-
ZDB-GENE-040426-929; B0R157_DANRE	Pex12	Ubiquitin-protein transferase activity. Involved in protein import into peroxisome matrix and protein mono-ubiquitination of Pex5. Localizes to peroxisomal importomer complex. RING Zinc finger, C3HC4 type?	n.a.	n.a.	-
ZDB-GENE-040426-1544; Q6PFQ3_DANRE	Pex13	Involved in protein import into peroxisome matrix, docking. Part of the peroxisomal membrane docking complex for Pex5 and Pex7. Localizes to peroxisomal importomer complex and peroxisomal membrane. SH3 domain.	n.a.	n.a.	-
ZDB-GENE-060130-169; A0A2R8QMZ2_DANRE	Pex14	Involved in protein import into peroxisome matrix, docking. Part of the peroxisomal membrane docking complex for Pex5 and Pex7. Localizes to peroxisomal importomer complex and peroxisomal membrane.	n.a.	n.a.	-
ZDB-GENE-050626-49; F1RDG2_DANRE	Pex16	Involved in protein import into peroxisome membrane. Recruitment of Pex3 to the peroxisome membrane, required for	n.a.	n.a.	-

		de novo formation. Involved in peroxisome organization. Localizes to peroxisomal membrane.			
ZDB-GENE-050417-424; F1R313_DANRE	Pex19	Involved in protein import into peroxisome membrane. Peroxisome membrane protein import receptor, required for *de novo* formation. Interacts with Pex3. Localises to cytosol and peroxisome membrane. CAAX-box (farnesylation site).	n.a.	n.a.	
ZDB-GENE-030131-6584; F1RBL0_DANRE	Pex26	Pex1/Pex6 anchor protein at the peroxisomal membrane. ATPase binding activity. Involved in protein import into peroxisome matrix. Recycling of Pex5. Tail-anchored membrane protein.	n.a.	n.a.	-
**Other proteins involved in peroxisome biogenesis**					
ZDB-GENE-050506-92; A0A2R8Q8G0_DANRE	Fis1	Involved in mitochondrial/peroxisomal division, membrane adaptor for Dnm1l. Tail-anchored membrane protein.	n.a.	n.a.	-
ZDB-GENE-040426-1510; F1Q877_DANRE	Mffa (mitochondrial fission factor)	Involved in mitochondrial/peroxisomal division, major membrane adaptor for Dnm1l. Tail-anchored membrane protein.	n.a.	n.a.	-
ZDB-GENE-050522-208; A8E7S0_DANRE	Mffb (mitochondrial fission factor)	Involved in mitochondrial/peroxisomal division, major membrane adaptor for Dnm1l. Tail-anchored membrane protein.	n.a.	n.a.	-
ZDB-GENE-040426-1556; A0A140LH30_DANRE	Dnm1l (Dynamin 1-like protein)	Involved in mitochondrial/peroxisomal fission. Member of the dynamin family of GTPases	n.a.	n.a.	-
ZDB-GENE-081105-41; F6P2T1_DANRE	si:dkey-32e23.4	Orthologous to human DNM1L	n.a.	n.a.	-
ZDB-GENE-030131-5354;	Rhot1a (Miro1)	Involved in mitochondrial/peroxisomal motility. Adaptor protein for kinesin. Tail-anchored membrane protein.	n.a.	n.a.	-

Q6NVC5_DANRE					
ZDB-GENE-061009-52; A0A0R4IGX0_DANRE	Rhot1b (Miro1)	Involved in mitochondrial/peroxisomal motility. Adaptor protein for kinesin. Tail-anchored membrane protein.	n.a.	n.a.	-
ZDB-GENE-051120-96; Q32LU1_DANRE	Rhot2 (Miro2)	Involved in mitochondrial/peroxisomal motility. Adaptor protein for kinesin. Tail-anchored membrane protein.	n.a.	n.a.	-
**Lipid metabolism – fatty acid activation/transport**					
ZDB-GENE-050706-104; F1QQC5_DANRE	Slc27a2a	Solute carrier family 27 member 2a. Predicted to have long-chain fatty acid transporter activity and very long/long-chain fatty acid-CoA ligase activity.	○ TRL[G]		-
ZDB-GENE-081104-49; Q1ECW0_DANRE	Slc27a2b	Solute carrier family 27 member 2b. Predicted to have long-chain fatty acid transporter activity and very long/long-chain fatty acid-CoA ligase activity.	○ FRL		
ZDB-GENE-050417-248; Q567D7_DANRE	Slc27a4 (zgc:112138)	Solute carrier family 27 member 2b. Predicted to have long chain- fatty acid transporter activity and very long-chain/long chain fatty acid-CoA ligase activity	● QKL[G]		-
ZDB-GENE-040912-169; F1RAK0_DANRE	Acsl5	Acyl-CoA synthetase long chain family member 5. Fatty acid activation by coupling to coenzyme A. Predicted to localize to integral component of membrane. N-tail anchored membrane protein.	○ ANM		-
ZDB-GENE-050517-27; F1RBC8_DANRE	Abcd1	ATP-binding cassette (ABC) sub-family D membrane transporter, fatty acid import	n.a.	n.a.	○
ZDB-GENE-050517-28; E7F973_DANRE	Abcd2	ATP-binding cassette (ABC) sub-family D membrane transporter, fatty acid import	n.a.	n.a.	-
ZDB-GENE-040426-2868; A0A0R4IRL4_DANRE	Abcd3a	ATP-binding cassette (ABC) sub-family D membrane transporter, fatty acid import	n.a.	n.a.	●
ZDB-GENE-050517-29;	Abcd3b	ATP-binding cassette (ABC) sub-family D membrane	n.a.	n.a.	○

B0UY91_DANRE		transporter, fatty acid import			
ZDB-GENE-050522-268; E9QCH6_DANRE; NP_001018483.1	Acbd5a	Acyl-CoA binding domain containing protein 5a. Fatty-acyl-CoA binding activity. Required for VLCFA-acyl-CoA import via ABCD1. Involved in peroxisome-ER tethering and membrane contact site formation. Tail-anchored membrane protein.	n.a.	n.a.	-
ZDB-GENE-070705-18; A5WV69_DANRE; NP_001093558.1	Acbd5b	Acyl-CoA binding domain containing protein 5b. Fatty-acyl-CoA binding activity (see above).	n.a.	n.a.	-
ZDB-GENE-040426-2074; F1QA31_DANRE; NP_998260.1	Acbd4	Acyl-CoA binding domain containing protein 4. Fatty-acyl-CoA binding activity. Involved in peroxisome-ER tethering and membrane contact site formation. Potential tail-anchored membrane protein.	n.a.	n.a.	-
**Lipid metabolism – fatty acid β-oxidation**					
ZDB-GENE-040426-814; F1RE17_DANRE	Acad11	Fatty acid β-oxidation, first step (?). Predicted to have flavin adenine dinucleotide binding activity and oxidoreductase activity, acting on the CH-CH group of donors. Predicted to be involved in oxidation-reduction process.	● AKL		-
ZDB-GENE-041010-219; F1R071_DANRE	Acox1	Acyl-CoA oxidase. Fatty acid β-oxidation, first step. Fatty acid binding activity. Flavin adenine dinucleotide binding activity. Palmitoyl-CoA oxidase activity.	● SKL		-
ZDB-GENE-081107-18; A0A2R8QDD9_DANRE ; F1R4J4_DANRE (no PTS1/PTS2)	Acox-like (acoxl) (isoform)	Predicted to have acyl-CoA oxidase activity; fatty acid binding activity and flavin adenine dinucleotide binding activity. Predicted to be involved in fatty acid beta-oxidation using acyl-CoA oxidase and lipid homeostasis.	● SKL	NO	-
ZDB-GENE-040426-2163; F1QXK3_DANRE	Acox3	Acyl-CoA oxidase. Fatty acid β-oxidation, first step. Fatty acid binding activity. Flavin adenine dinucleotide binding activity.	● AKL		-

		Pristanoyl-CoA oxidase activity.			
ZDB-GENE-040426-2581; Q6NYL3_DANRE	Ehhadh (L-BP)	Enoyl-CoA hydratase/3-hydroxyacyl CoA dehydrogenase. Fatty acid β-oxidation, second & third step	● SHL		●
ZDB-GENE-040421-1; Q6NZW5_DANRE	Hsd17b4 (D-BP)	Hydroxysteroid (17-beta) dehydrogenase 4. Fatty acid β-oxidation, second & third step.	● ARL		-
ZDB-GENE-040704-48; Q6GQN6_DANRE	Acaa1	Peroxisomal 3-ketoacyl-CoA thiolase. Acetyl-CoA C-acyltransferase. Fatty acid β-oxidation, fourth step.		● RVNIL SGHL	●
ZDB-GENE-040426-1846; Q6P4V5_DANRE	Scp2a	Sterol carrier protein 2a (SCPX) (538aa). Thiolase domain. Fatty acid β-oxidation, fourth step.	● AKL		●
ZDB-GENE-041010-196; Q5XJS3_DANRE	Scp2b	Sterol carrier protein 2b (142aa). Truncated from lacking the thiolase domain.	● AKL		●
ZDB-GENE-050522-484; A0A2R8QEJ3_DANRE	Amacr	α-Methylacyl-CoA racemase. β-oxidation of 2-methyl branched-chain fatty acids. Conversion of pristanoyl-CoA and C27-bile acyl-CoA to (S)-stereoisomers. Predicted to have CoA-transferase activity.	● ARL		●
ZDB-GENE-041010-174; Q5XJP9_DANRE	Acot8	Peroxisomal acyl-CoA thioesterase 8. Predicted to have acyl-CoA hydrolase activity. Predicted to be involved in acyl-CoA metabolic process and fatty acid catabolic process.	● SKL		-
ZDB-GENE-050417-232; Q567C7_DANRE	Pecr	Peroxisomal trans-2-enoyl-CoA reductase. Predicted to have trans-2-enoyl-CoA reductase (NADPH) activity. Predicted to be involved in oxidation-reduction process and phytol metabolic process.	● SKL		●
ZDB-GENE-050522-35; F1QAI1_DANRE	Crot	Carnitine O-octanoyltransferase	● SQL[G]		-
ZDB-GENE-040927-17;	Crata	Carnitine O-acetyltransferase a. Export of fatty acids from	● AKL		●

Q642H6_DANRE		peroxisomes			
Not present in D. rerio	BAAT	Bile acid-CoA:amino acid N-acyltransferase	-	-	-
**Lipid metabolism – degradation of polyunsaturated fatty acids**					
ZDB-GENE-040426-2612; Q6NV34_DANRE	Decr2	Peroxisomal 2,4-dienoyl-CoA reductase 2. Catalyzes the reduction of 2,4-dienoyl-CoA to trans-3-enoyl-CoA. 2,4-dienoyl-CoA reductase (NADPH) activity.	● AKL		-
ZDB-GENE-040718-392; A0A0R4IAH3_DANRE	Eci2	Δ3,Δ2-enoyl-CoA isomerase (PECI). Isomerization of 3-trans,5-cis-dienoyl-CoA to 2-trans,4-trans-dienoyl-CoA.	● SKL		●
ZDB-GENE-041010-170; F1Q8H6_DANRE	Ech1	Peroxisomal enoyl CoA hydratase 1 (ECH1). Isomerization of 3-trans,5-cis-dienoyl-CoA to 2-trans,4-trans-dienoyl-CoA.	● SKL		●
ZDB-GENE-041010-72; F1R2G5_DANRE	zgc:101569	Enoyl-CoA hydratase/isomerase family.	● SKL		●
**Lipid metabolism – fatty acid α-oxidation**					
ZDB-GENE-050417-361; E7FCS1_DANRE	Phyh	Phytanoyl-CoA 2-hydroxylase. Predicted to have phytanoyl-CoA dioxygenase activity.		● RLKVVLNHL	-
ZDB-GENE-040426-2058; F1RB36_DANRE	Hacl1	2-hydroxyacyl-CoA lyase 1. Conversion of 2-hydroxyphytanoyl-CoA to pristanal.	● SNL		-
ZDB-GENE-040718-74; A0A2R8PW97_DANRE	Aldh3a2a	Fatty aldehyde dehydrogenase. Conversion of pristanal to pristanic acid. Tail-anchored membrane protein.	n.a.	n.a.	●
ZDB-GENE-040912-103; E9QH31_DANRE	Aldh3a2b	Fatty aldehyde dehydrogenase. Conversion of pristanal to pristanic acid. Tail-anchored membrane protein.	n.a.	n.a.	-
**Lipid metabolism – miscellaneous proteins**					
ZDB-GENE-050417-	Pecr	Peroxisomal trans-2-enoyl-CoA reductase. Predicted to have	● SKL		●

232; Q567C7_DANRE		trans-2-enoyl-CoA reductase (NADPH) activity. Predicted to be involved in oxidation-reduction process and phytol metabolic process.			
ZDB-GENE-070410-120; A3KP65_DANRE	Mlycd	Malonyl-CoA decarboxylase. Involved in acetyl-CoA biosynthetic process, malonyl-CoA catabolic process and positive regulation of fatty acid oxidation	● SKL		●
**Lipid metabolism – etherphospholipid synthesis**					
ZDB-GENE-040618-4; F1QAU7_DANRE	Gnpat	Glycerone-phosphate O-acyltransferase. Synthesis of acyl-DHAP from DHAP and acyl-CoA.	● ARL		-
ZDB-GENE-030131-8374; B8JM22_DANRE; A0A2R8QK16_DANRE	si:ch73-21k16.5 Gnpat2	Predicted to have glycerone-phosphate O-acyltransferase activity.	● ARL ● SVL		
ZDB-GENE-040426-2908; A0A0R4ICF6_DANRE	Far1	Fatty acyl CoA reductase 1. Fatty alcohol synthesis. Putative tail-anchored membrane protein.	n.a.	n.a.	-
ZDB-GENE-040426-2908; F1QSU9_DANRE	Far1 (isoform)	Fatty acyl CoA reductase 1. Fatty alcohol synthesis.	● SRL		-
ZDB-GENE-060503-367; Q1L8Q4_DANRE	si:dkey-97m3.1 (Far2)	Predicted to have fatty-acyl-CoA reductase (alcohol-forming) activity. Predicted to be involved in long-chain fatty-acyl-CoA metabolic process. Orthologous to human FAR2 (fatty acyl-CoA reductase 2). Putative tail-anchored membrane protein.	n.a.	n.a.	-
ZDB-GENE-031118-14; F1QC72_DANRE	Agps	Alkylglycerone-phosphate synthase. Predicted to have FAD binding activity and oxidoreductase activity.		● RLRIIAGHL	-
ZDB-GENE-050417-277; F1QWU6_DANRE	Dhrs7b	Dehydrogenase/reductase SDR family member 7B. Acyl/alkyl-DHAP reductase. Putative N-tail-anchored membrane protein.	n.a.	n.a.	-

**Glycolate/glyoxylate metabolism**					
ZDB-GENE-060519-2; Q7SXE5_DANRE	Hao1	2-Hydoxyacid oxidase 1. Glycolate oxidase activity. Converts glycolate into glyoxylate.	● SRI		
ZDB-GENE-040426-1239; F1QCD8_DANRE	Hao2 (long chain)	Hydoxyacid oxidase 2. Predicted to have FMN binding activity and oxidoreductase activity.	● SRL		-
ZDB-GENE-130530-761; A0A0R4IAX5_DANRE	Mlsl	Malate synthase-like. Acetyl-CoA + H_2O + glyoxylate = (S)-malate + CoA	● ARL		-
ZDB-GENE-040718-16; F1QY24 _DANRE	Agxta	Alanine-glyoxylate and serine-pyruvate aminotransferase. Predicted to have alanine-glyoxylate transaminase activity and serine-pyruvate transaminase activity. Predicted to be involved in glycine biosynthetic process, by transamination of glyoxylate.	● SRV		-
**Amino acid catabolism**					
ZDB-GENE-050913-127; Q4V981_DANRE; A2BGT3_DANRE	Dao1<br>Dao1, tandem duplicate 1	D-amino-acid oxidase 1. Degradation of D-amino acids.	● SRL		-
ZDB-GENE-040426-2634; Q6NY97_DANRE; A2BGT2_DANRE	Dao2<br>Dao2, tandem duplicate 2	D-amino-acid oxidase 2. Degradation of D-amino acids.	● SRL		-
ZDB-GENE-040426-1894; Q6P009_DANRE	Dao3	D-amino-acid oxidase 3. Degradation of D-amino acids.	● SRL		-
ZDB-GENE-080204-116; A0A0R4IZE6_DANRE	zgc:172341(Ddo)	D-aspartate oxidase. Degradation of aspartate.	● ARL		-
ZDB-GENE-040426-	Hmgcl	Hydroxymethylglutaryl-CoA lyase. (3S)-hydroxy-3-	● CKL		●

958; F1QTF0_DANRE		methylglutaryl-CoA = acetoacetate + acetyl-CoA (ketogenesis). Terminal step in leucine catabolism.			
ZDB-GENE-110627-2; A7MBQ2_DANRE; F6P928_DANRE	Pipox	Pipecolic acid oxidase. Oxidation of L-pipecolate and L-proline. Predicted to have L-pipecolate oxidase activity and sarcosine oxidase activity. Predicted to be involved in L-lysine catabolic process to acetyl-CoA via L-pipecolate.	● SSL		
**Polyamine oxidation**					
ZDB-GENE-090312-204; B8JJQ4_DANRE	si:dkey-275b16.2 (Paox)	Polyamine oxidase (peroxisomal N(1)-acetyl-spermine/spermidine oxidase). Regulation of intracellular polyamine concentration	● SKL		-
**Purine and pyrimidine metabolism**					
ZDB-GENE-030826-24; Q6DG85_DANRE	Uox	Urate oxidase (uricase). Conversion of uric acid to allantoin $H_2O + O_2$ + urate = 5-hydroxyisourate + H_2O_2	● ARM		-
ZDB-GENE-040718-471; Q6DGA6 (ALLC_DANRE) B2GQF5_DANRE	Allc	Allantoicase. Allantoate + H_2O (S) = ureidoglycolate + urea	● SKL		-
ZDB-GENE-060825-253; A0A0R4IGA6_DANRE	Uraha	Urate (5-hydroxyiso-) hydrolase a. 5-hydroxyisourate + H_2O = 5-hydroxy-2-oxo-4-ureido-2,5-dihydro-1*H*-imidazole-5-carboxylate + H^+ Exhibits hydrolase activity, acting on carbon-nitrogen (but not peptide) bonds, in linear amides and hydroxyisourate hydrolase activity. Involved in purine nucleobase metabolic process.		● RLQHI RGHIV	-
ZDB-GENE-070112-472; A1L259 (URAD_DANRE)	Urad	Ureidoimidazoline decarboxylase. 5-hydroxy-2-oxo-4-ureido-2,5-dihydro-1*H*-imidazole-5-carboxylate + H^+ = (*S*)-allantoin + CO_2	● TKL^G		-

		Exhibits 2-oxo-4-hydroxy-4-carboxy-5-ureidoimidazoline decarboxylase activity. Predicted to be involved in allantoin biosynthetic process; purine nucleobase metabolic process; and urate catabolic process.			
**Oxygen metabolism and oxidation of redox equivalents**					
ZDB-GENE-000210-20; A0A0R4II89_DANRE; Q9PT92_DANRE	Cat	Catalase. Heme binding activity.	● SKM		-
ZDB-GENE-041212-70; F1R264_DANRE	Ephx2	Epoxide hydrolase 2.	● PKL		-
ZDB-GENE-040718-298; Q6DGU9_DANRE	Gstk1	Glutathione S-transferase kappa 1. RX + glutathione = HX + R-S-glutathione	● AKM		-
ZDB-GENE-050417-322; A0A0R4IHZ8_DANRE	Ccs	Copper chaperone for superoxide dismutase. Predicted to be involved in removal of superoxide radicals.	● SHL		-
ZDB-GENE-990415-258; O73872 (SODC_DANRE)	Sod1	Superoxide dismutase [Cu-Zn]. Removal of superoxide radicals. 2 H^+ + 2 superoxide = H_2O2 + O_2 (imported piggy-back into peroxisomes via Ccs)	NT		-
ZDB-GENE-050522-159; F1QCE3_DANRE	Prdx5	Peroxiredoxin 5. Reduction of hydrogen peroxide and alkyl hydroperoxides. Predicted to have thioredoxin peroxidase activity. Involved in fin regeneration (peroxisomal localisation?)	NT	NT	●
**Proteases**					
ZDB-GENE-070410-85; A0A0R4IL71_DANRE	Ide	Insulin-degrading enzyme. Predicted to have metal ion binding activity and metallo-endopeptidase activity.	● AKL		●
ZDB-GENE-041212-1; A0A0R4ISE4_DANRE	Lonp2	Lon Protease homolog 2. Degradation of misfolded or damaged polypeptides in peroxisomes. Predicted to have ATP binding	● SKL		-

		activity, ATP-dependent peptidase activity, and serine-type endopeptidase activity.			
ZDB-GENE-030131-8525; B3DFU6_DANRE	Tysnd1	Trypsin domain containing 1. Predicted to have serine-type endopeptidase activity. Predicted to be involved in protein processing and regulation of fatty acid beta-oxidation.	● SKL		-
**Carbohydrate metabolism, NADP+/NADPH and NAD+/NADH Shuttle**					
ZDB-GENE-070508-4; F6NHI0_DANRE	G6pd	Glucose 6-phosphate dehydrogenase. D-glucose 6-phosphate + NADP+ = D-glucono-1,5-lactone 6-phosphate + NADPH + H+	● HKL		-
ZDB-GENE-031006-1; B0UXL2_DANRE	Idh1	Isocitrate dehydrogenase 1 (NADP(+). Isocitrate + NADP+ = 2-oxoglutarate + CO_2 + NADPH	● PKL		-
ZDB-GENE-040204-1; NP_001303854.1 isoform mdh1x	Mdh1aa isoform	Malate dehydrogenase 1Aa. L-malate dehydrogenase activity. Predicted to be involved in NADH metabolic process; dicarboxylic acid metabolic process, and tricarboxylic acid cycle. C-terminally extended isoform generated by translational read-through.	● SRL		-
**Cleavage of Cofactors**					
ZDB-GENE-131127-212; E7FAS2_DANRE	Nudt7	Nucleoside diphosphate-linked moiety X motif 7. CoA diphosphatase (NUDT7). Acetyl-CoA hydrolase activity. Regulation of CoA and acyl-CoA levels in response to metabolic demands.	● SKL		-
ZDB-GENE-050417-164; Q567I5_DANRE	Nudt12	NADH pyrophosphatase NUDT12. Regulation of NADH levels in response to metabolic demands.	NT		-
ZDB-GENE-081022-80; B3DHE6_DANRE	Nudt19	Nucleoside diphosphate-linked moiety X motif 19. Nudix hydrolase 19 (NUDT19/RP2). Regulation of CoA and acyl-CoA levels in response to metabolic demands.	● SKL		-
**Miscellaneous peroxisomal membrane proteins**					

ZDB-GENE-070620-4; A5D6T2_DANRE	Slc25a17 (PMP34) Zgc:162641	Peroxisomal CoA transporter. Multi-pass membrane protein.	n.a.	n.a.	-
ZDB-GENE-070620-4	Slc25a17l (PMP34-like) Zgc:162641	Slc25a17-like. Peroxisomal CoA transporter. Multi-pass membrane protein.	n.a.	n.a.	
ZDB-GENE-040912-184; Q66HU7_DANRE	Pxmp2	Peroxisomal membrane protein 2 (PMP22). Potential pore-forming protein.	n.a.	n.a.	●
ZDB-GENE-030131-4872; A0A2R8QFW3_DANRE	Pxmp4	Peroxisomal membrane protein 4 (PMP24). Peroxisomal metabolite transport?	n.a.	n.a.	●
ZDB-GENE-070424-70; A4QN71_DANRE	Tmem135	Transmembrane protein 135 (PMP52). Peroxisomal metabolite transport? Multi-pass membrane protein.	n.a.	n.a.	-
ZDB-GENE-040426-1168; Q5TZ51_DANRE	Mpv17	Involved in iridophore differentiation. Multi-pass membrane protein. Mitochondrial inner membrane. Peroxisomal?	● NKM		●
ZDB-GENE-120215-229; A0A0R4IUD3_DANRE	Mpv17l Si:dkeyp-72e1.7	Mpv17 like. Multi-pass membrane protein. Peroxisomal?	n.a.	n.a.	●
ZDB-GENE-040718-306; Q6DGV7_DANRE	Mpv17l2	MPV17-like 2. Multi-pass membrane protein. Peroxisomal?	n.a.	n.a.	●
ZDB-GENE-050327-95; A0A2R8PWS6_DANRE	Marc1	Mitochondrial amidoxime reducing component 1 (MARC1/MOSC1). Reduction of N-hydroxylated drugs in mitochondria. Mitochondria. Peroxisomal localisation?	n.a.	n.a.	-
ZDB-GENE-070112-1402; F1REK4_DANRE	Mavs	Mitochondrial antiviral-signaling protein. Putative tail-anchored membrane protein.	n.a.	n.a.	-
ZDB-GENE-030616-593; A0A2R8RM20_DANRE	Atad1a	Predicted to have ATP binding activity and ATPase activity. Orthologous to human ATAD1 (ATPase family AAA domain containing 1). Putative tail-anchored membrane protein.	n.a.	n.a.	-

ZDB-GENE-030616-44; B2GP29_DANRE	Atad1b	Predicted to have ATP binding activity and ATPase activity. Orthologous to human ATAD1 (ATPase family AAA domain containing 1). Putative tail-anchored membrane protein.	n.a.	n.a.	-
ZDB-GENE-060526-335; A2BGT0 (UBP30_DANRE)	Usp30	Ubiquitin carboxyl-terminal hydrolase 30. Membrane protein. Mitochondria and peroxisomes in mammals.	n.a.	n.a.	-
ZDB-GENE-091118-62; A0A0R4IKJ8_DANRE	Slc22a21	Solute carrier family 22 member 21. Organic cation transporter (Octn3). Transmembrane protein. Carnitine transport into peroxisomes?	n.a.	n.a.	-
ZDB-GENE-030131-2957; E7FBZ8_DANRE	Trim37	Tripartite motif-containing 37. E3 ubiquitin-protein ligase. Peroxisomal protein import/receptor recycling.	n.a.	n.a.	-
ZDB-GENE-070705-553; F1RE62_DANRE	Pnpla8	Patatin-like phospholipase domain-containing 8. Calcium-independent phospholipase A2-gamma. Promotes cellular membrane hydrolysis. Participates in the generation of lipid second messengers. Putative membrane protein.	● SKL	n.a.	-
ZDB-GENE-060503-916; G1K2P4_DANRE	fndc5b (isoform)	Fibronectin type III domain containing 5b (PeP/GNDC5). Predicted to have hormone activity. Predicted to also localize to extracellular region and plasma membrane. Potential role in myoblast differentiation and development. Single-pass membrane protein.	● SKV	n.a.	-
**Ascorbate biosynthesis**					
Absent in teleost fish and humans	Gulo	L-gulonolactone oxidase. Oxidizes L-gulono-1,4-lactone to H_2O_2 and L-xylo-hexulonolactone. Ultimate enzyme of hepatic ascorbate formation in ascorbate-synthesizing species. Localises to ER and peroxisomes in mouse liver. Single pass membrane protein.	-	-	-

**Other PTS1-containing proteins with unknown function**					
ZDB-GENE-030131-1368; A2BGU9 (SERHL_DANRE)	serhl	Serine hydrolase-like protein	● SRL		●
ZDB-GENE-040426-1498; A0A2R8Q976_DANRE; F1Q8N0_DANRE	Dhrs4	Dehydrogenase/reductase (SDR family) member 4. Oxidoreductase activity. Puative Short-chain alcohol dehydrogenase. Retinol metabolism. Predicted TMD and membrane localisation. F1Q8N0 lacks TMD.	● SRL		-
ZDB-GENE-100208-2; F1RDC7_DANRE	Usp2b	Ubiquitin carboxyl-terminal hydrolase. Short isoform XP_009300198.1 (the long isoform XP_005157570.1 lacks a PTS1)	● SRM		-
ZDB-GENE-061020-1; Q08C33 (ISOC1_DANRE)	Isoc1	Isochorismatase domain containing 1	● SKV		-
ZDB-GENE-040426-2257; Q6NYF0_DANRE	Lactb2	β-Lactamase-like protein 2. Endoribonuclease activity; single-stranded RNA binding activity; and zinc ion binding activity. Localizes to mitochondria in humans.	● SNL		●
ZDB-GENE-030131-1066; Q6P5L8_DANRE	Hsdl2	Hydroxysteroid dehydrogenase-like 2. Oxidoreductase activity. Involved in oxidation-reduction process.	● SKL		●
ZDB-GENE-040912-137; Q66HW0_DANRE	CoA synthase (coasy)	Dephospho-CoA kinase activity and pantetheine-phosphate adenylyltransferase activity. Involved in coenzyme A biosynthetic process. Localises to mitochondria in humans.	● SSL		

PTS1/2, peroxisomal targeting signal 1/2; MTS, N-terminal mitochondrial targeting signal; n.a. not applicable; NT, not targeted

● - predicted strong targeting signal, ● - predicted weak targeting signal (twilight zone), [G] - Using general prediction function
